# Combining 5-ALA-PDT with berbamine as an in vitro multimodal therapy approach against bladder cancer cells

**DOI:** 10.1038/s41598-026-46092-x

**Published:** 2026-04-01

**Authors:** Muriel Kabus, Maximilian Aumiller, Adrain Rühm, Thomas Pongratz, Michèle J Hoffmann, Alexander Buchner, Ronald Sroka, Heike Pohla

**Affiliations:** 1 Laboratory of Tumor Immunology, LIFE Center, LMU University Hospital, LMU Munich, Fraunhoferstr. 20, 82152 Planegg, Germany; 2 Laser Research Laboratory, LIFE Center, LMU University Hospital, LMU Munich, Fraunhoferstr. 20, 82152 Planegg, Germany; 3 Department of Urology, LMU University Hospital, LMU Munich, Marchioninistr. 15, 81377 Munich, Germany; 4 Department of Urology, Medical Faculty and University Hospital Duesseldorf, Heinrich Heine University Duesseldorf, Moorenstr. 5, 40225 Duesseldorf, Germany

**Keywords:** Bladder cancer, 5-ALA-mediated photodynamic therapy, Protoporphyrin IX, Berbamine, Cancer stem cells, Cisplatin resistance, Cancer, Drug discovery, Oncology

## Abstract

Bladder cancer is a common urogenital malignancy that remains difficult to treat, particularly due to therapeutic resistance, such as resistance to cisplatin, in which cancer stem cells (CSCs) play a central role. This study investigates the combination of 5-aminolevulinic acid-mediated photodynamic therapy (5-ALA-PDT) and berbamine as a potential multimodal treatment strategy using the bladder cancer cell lines RT112 and J82, their cisplatin-resistant variants, and generated CSC-like cells. Berbamine is a natural plant compound and was confirmed in this study to have anticancer properties by inhibiting cell migration and invasion, and by inducing apoptosis. This study also showed that berbamine enhances the accumulation of protoporphyrin IX (PpIX), the photosensitizer induced by 5-ALA. 5-ALA-PDT destroys cancer cells by stimulating PpIX via 635 nm red laser light to produce reactive oxygen species (ROS). This was found to happen in all tested cell lines, whereas berbamine could modulate the cell destruction in a concentration-dependent manner and was influenced by the specific biological characteristics of the tested cell variants. CSCs showed the strongest response to the combination therapy approach, suggesting that they may represent more vulnerable cell variants to the tested treatment. Cisplatin-resistant cell lines could also be treated successfully with 5-ALA-PDT, whereas berbamine could enhance its efficacy in the cisplatin-resistant J82 LTT. These findings suggest that the combination treatment of 5-ALA-PDT and berbamine may serve as a promising approach to overcome therapeutic resistance in bladder cancer, particularly in cisplatin-resistant and CSC-enriched tumour types.

**Supplementary Information:**

The online version contains supplementary material available at 10.1038/s41598-026-46092-x.

## Introduction

Bladder cancer remains a global health concern. According to GLOBOCAN 2022 estimates, it ranked ninth in terms of incidence and thirteenth in terms of mortality worldwide, accounting for approximately 614,000 new cases and 220,000 deaths in 2022^1^. While more than two-thirds of cases occur in men, the disease remains a substantial concern across both sexes^1,2^. Incidence and mortality rates are disproportionately higher in high-income countries, particularly in Europe^1^. The most relevant bladder cancer risk factors besides sex are age (median age at diagnosis: 77 a for women, 75 a for men in Germany in the year 2020), tobacco use, and exposure to carcinogenic chemicals such as aromatic amines^3^.

Bladder cancer is clinically classified into three main categories: non-muscle-invasive bladder cancer (NMIBC), muscle-invasive bladder cancer (MIBC), and metastatic disease. According to the NCCN Guidelines®, NMIBC represents approximately 75% of all newly diagnosed cases and is typically managed with intravesical therapies, transurethral resection, and Bacillus Calmette Guérin (BCG) treatment, with the primary goal of reducing recurrence and progression. MIBC requires a more aggressive, multimodal approach, often including radical cystectomy, systemic chemotherapy, and/or radiation. Metastatic bladder cancer occurs in 5% of cases and is associated with a poor prognosis. It is treated as first-line with chemotherapeutics such as cisplatin, or alternatively, immune checkpoint inhibitors, antibody–drug conjugates, and targeted therapies^4^. Recurrences are a major problem in the treatment of bladder cancer^5^. Even for low-grade NMBIC, which has a low tendency to progress, more than 40% of the cases show recurrence within the first 5 years after treatment^6^. Therefore, once diagnosed with cancer, patients must undergo lifelong treatment or monitoring using a combination of cystoscopy, imaging, and urine cytology^7^. Thus, health care for bladder cancer patients is an economic burden worldwide and costs the EU €4.9 billion in 2012 and nearly $4 billion in the US in 2010^8,9^.

Cisplatin-based chemotherapy remains a basis treatment of advanced bladder cancer, both in the neoadjuvant and metastatic setting, provided that patients meet strict eligibility criteria related to renal function and performance status^4,10^. Unfortunately, the emergence of resistance to cisplatin is a common and often unavoidable challenge during treatment, whereas the resistance mechanisms are quite multifaceted. Cisplatin resistance involves reduced drug influx via CTR1^11,12^, increased efflux through e.g., MRP-1 and GS-X pump, as well as enhanced detoxification by glutathione and GST^13^, or epigenetic mechanisms such as DNA methylation, non-coding RNAs, and post-translational modifications^14^.

Among the various mechanisms implicated in cisplatin resistance, accumulating evidence suggests a critical role for cancer stem cells (CSCs). These cells represent a small subpopulation of tumour cells characterised by their ability of self-renewal, unlimited proliferation, and differentiation^15,16^. Their properties are closely associated with increased recurrence rates, enhanced tumour growth, higher metastatic potential, resistance to various therapeutic approaches, and overall poorer clinical outcomes than differentiated tumour cells^17,18^. Bladder CSCs were first identified in 2009 as tumour-initiating cells, isolated based on their expression of normal stem cell markers such as CD44. This surface protein plays a key role in regulating cancer cell proliferation, differentiation, migration, angiogenesis, and overall disease progression^19^. Aldehyde dehydrogenase 1 A1 (ALDH1A1) was found to be another key marker in bladder CSCs and is associated with tumour progression, recurrences, and poor prognosis^20,21^. In light of the complex resistance mechanisms, therapeutic strategies targeting bladder CSCs are urgently needed. One such promising approach is 5-aminolaevulinic acid-mediated photodynamic therapy (5-ALA-PDT)^22^, which combines selective cellular uptake with localised ROS-mediated oxidative damage and has shown potential to target CSCs in reducing ALDH1 activity and CD44 positivity in head and neck CSCs that show cisplatin resistance^23^. Previous studies reported greater 5-ALA-PDT sensitivity in glioblastoma CSCs compared to their parental cells, likely due to higher expression of protoporphyrin IX (PpIX) biosynthesis enzymes and transporters such as PEPT1/2 and ABCD6^24^.

Furthermore, 5-ALA-PDT has gained considerable interest as a targeted treatment modality in bladder cancer^22^. 5-ALA is a metabolite in the haem biosynthesis pathway that, through various precursors in the cytoplasm, leads to production of PpIX in mitochondria^25^, but also can diffuse in other cellular membrane sites^26^. PpIX serves as the immediate precursor of haem and is converted by the mitochondrial enzyme ferrochelatase^25^. It acts as a photosensitiser and, upon activation by light of specific wavelengths, generates reactive oxygen species (ROS), which induce oxidative stress, leading to necrosis, apoptosis, and autophagy in tumour cells^27,28^. Upon exogenous administration of 5-ALA, intracellular accumulation of PpIX can occur due to bypass feedback inhibition and the limited catalytic capacity of ferrochelatase^29^. In tumour cells, this accumulation is typically enhanced by elevated activity of porphobilinogen deaminase and decreased activity of ferrochelatase, resulting in preferential PpIX accumulation^30^. The activity of efflux transporters, including ABCG2, can contribute to altered PpIX accumulation^31^. Studies on bladder cancer cell lines showed that the PpIX content was 9 to 16 times higher than in normal urothelial cells^32^. Thus, resulting in tumour-specific accumulation of PpIX, which can be further activated by applying light via a thin optical fibre into the urinary bladder to illuminate the tumour site^33^. The first clinical trials conducted in 1996 using intravesically applied 5-ALA and red light illumination at a wavelength of 635 nm with energy doses of up to 60 J/cm^2^ demonstrated high remission rates in patients with bladder cancer^22^. While 5-ALA-PDT has shown promising results in clinical trials for NMBIC, especially for those who failed BCG therapy^34^, it is not yet established as a standard treatment in clinical practice^4,10,35^.

One of the main limitations of 5-ALA-PDT on cancer cells may lie in their antioxidant defence systems that counteract ROS-induced cytotoxicity^36^. Berbamine is a natural bis-benzylisoquinoline alkaloid isolated from the root of *Berberis amurensis* and has emerged as a promising candidate to affect these protective systems by inhibiting the Nuclear Factor κB (NF-κB) signalling pathway^37^. Berbamine is weakly cytotoxic in normal cells, but can stop tumour growth and support apoptosis in various cancer entities and interfere with several oncogenic cell signalling pathways^37,38^. In vivo studies have demonstrated the potential of berbamine as an enhancer in zinc phthalocyanine-based 5-ALA-PDT^39^. Such findings highlight that there is a medical need to enhance the efficacy of 5-ALA-PDT by identifying and validating effective sensitising agents like berbamine to develop combinatory approaches.

To address this, the present study investigated the effect of 5-ALA-PDT in combination with berbamine in selected bladder cancer cell lines. It focused particularly on cisplatin-resistant and CSC-enriched variants, which are known to drive recurrence and treatment failure. Also, it was examined whether berbamine influences PpIX accumulation, thereby potentially supporting 5-ALA-PDT efficacy. Supplementary examinations showed the effects of berbamine on cell migration, invasion, and apoptosis, as well as the impact of berbamine and 5-ALA-PDT on ROS generation.

## Materials and methods

### Cell culture, general cell handling procedures, and sphere formation

RT112 (DSMZ, cat. no. ACC-418) and J82 (ATCC, cat. no. HTB-1) cell lines originate from transitional cell carcinoma of the human bladder. They have been described to form tumours in nude mice and were verified via authentication testing (Eurofins Genomics; Luxembourg, Luxembourg). RT112 (RRID: CVCL_1670) was originally established in 1973 from an untreated tumour of histological grade G2 derived from a Caucasian female patient of unknown age^40^. J82 (RRID: CVCL_0359) was isolated from a 58-year-old Caucasian male patient^41^. RT112 and J82 cells were cultured in RPMI (Roswell Park Memorial Institute) 1640 medium (ThermoFisher, Carlsbad, CA, USA), supplemented with 10% foetal bovine serum (FBS; Biosell, Ennigerloh, Germany), 1% minimum essential medium non-essential amino acids (MEM NEAA; ThermoFisher), 1 mM sodium pyruvate (ThermoFisher), and 2 mM L-glutamine (ThermoFisher).

The cisplatin-resistant sublines RT112 LTT (long-term treated) and J82 LTT (both provided by PD Dr. Michéle J. Hoffmann of Heinrich Heine University Duesseldorf) were established by gradual cisplatin exposure. These cells were maintained in Dulbecco’s Modified Eagle Medium (DMEM) GlutaMAX™ (4.5 g/l D-glucose; ThermoFisher), supplemented with 10% FBS and 1 mM sodium pyruvate. Unless otherwise specified, this formulation was used in experiments for all cell lines and is hereafter referred to as experimental culture medium. To ensure stable resistance, RT112 LTT and J82 LTT cells were continuously cultured under selective pressure with cisplatin (Selleckchem, Houston, TX, USA) at final concentrations of 15.5 µg/ml cisplatin (51.5 µM) and 2.5 µg/ml (8.3 µM), respectively, throughout all experimental procedures.

In experimental settings with 5-ALA (Fagron, Rotterdam, Netherlands), 5-ALA was freshly prepared by dissolving 25 mg in 1 ml PBS, adjusting the pH to 7.4 with NaOH (Carl Roth, Karlsruhe, Germany), and filtering through a 0.22 µm membrane filter (Merck, Darmstadt, Germany). From this point onward, all procedures were conducted in the dark to prevent phototoxic effects. Berbamine (Selleckchem) stock solution of 50 mM was prepared by dissolving 1 g of berbamine powder in 29.34 ml dimethyl sulfoxide (CryoSure-DMSO, > 99.9% DMSO; WAK-Chemie, Steinbach, Germany). It had been shown, that the concentration of DMSO in the final dilution of performed cell culture experiments had no impact on the cell viability (unpublished results).

Sphere formation: For the enrichment of CSC-like cells RT112 and J82 were harvested with StemCell Accutase® (ThermoFisher), seeded to 10^6^ cells in 75 cm^2^ ultra-low attachment flasks (Corning, NY, USA), and cultured in DMEM and Ham’s F12 (DMEM/F12; ThermoFisher) supplemented with 2% of B-27 (ThermoFisher), 10 ng/ml basic fibroblast growth factor (bFGF; Sigma-Aldrich, St. Louis, MO, USA), and 10 ng/ml epidermal growth factor (EGF; Sigma-Aldrich) without serum. After 7 days of culture, spheres of RT112 CSC and J82 CSC were dissociated with StemCell Accutase®, and filtered to obtain a single-cell suspension for downstream applications.

All cell lines were maintained at 37 °C in a humidified incubator with 5% CO_2_. Detailed information on cell culture, general cell handling procedures, and sphere formation, as well as a general examination of morphological and proliferative characteristics, can be found in the supplementary section (1.; Supplementary Figs. S1, S2).

### Parameter optimization for combination therapy approach with 5-ALA-PDT and berbamine

To ensure optimal conditions for the combination treatment with 5-ALA-PDT and berbamine, key parameters such as cell seeding density and applied berbamine concentration were optimized based on the doubling time and IC_50_ values of each cell line. To present a broader understanding of the functional effects of the combined 5-ALA-PDT and berbamine treatment, detailed protocols, pre-experiments and resulting graphs are provided in the supplementary section (quantification of cell doubling time (2.; Supplementary Fig. S3), determination of IC50 berbamine and cisplatin (3.; Supplementary Figs. S4, S5), characterization of bladder cancer cell properties in relation to berbamine, such as cell migration (4.1; Supplementary Fig. S6), cell invasion (4.2.; Supplementary Fig. S7) and apoptosis rate (4.3.; Supplementary Figs. S8, S9), as well as determination of ROS generation following treatment with berbamine (5.1.; Supplementary Fig. S10) or 5-ALA-PDT (5.2.; Supplementary Fig. S11)).

#### Cell doubling time

To determine cell proliferation rates, cells were seeded at 1.6 × 10^4^ cells/cm^2^ in experimental culture medium into 25 cm^2^ culture flasks (ThermoFisher). Cisplatin was added to the medium for resistant cell lines to maintain selective pressure. After incubation at 37 °C and 5% CO_2_ for 48 h and 96 h, cells were counted under a light microscope, and doubling times were calculated based on the exponential Malthusian growth model using a statistical software (GraphPad Prism v10.2.3, GraphPad Software, San Diego, CA, USA; results see Table 1)^42^.

**Table 1:** Doubling time (h) and specified IC_50_ berbamine values (µM) for each cell line to ensure optimal conditions for combination treatment with 5-ALA-PDT and berbamine.

Cell line	Doubling time (h)	IC_50_ berbamine values (µM) for combination treatment
RT112	24.0	9.5
RT112 LTT	60.1	7.5
RT112 CSC	31.3	10.3
J82	44.8	10.7
J82 LTT	45.9	5.9
J82 CSC	47.3	13.6

#### Determination of IC_50_ berbamine

The half-maximal inhibitory concentration (IC_50_) of berbamine was determined following the experimental conditions used in the combination therapy approach with 5-ALA-PDT (see section below), to ensure a reliable correlation between berbamine concentration and cell viability across all bladder cancer cell lines. Cells were seeded as triplicate at a density of 15 × 10^3^ cells per well in 96-well plates (TPP Techno Plastic Products AG, Trasadingen, Switzerland) using 150 µl experimental culture medium. RT112 and RT112 LTT were seeded at 7.5 × 10^3^ and 22.5 × 10^3^ cells per well, respectively, according to their doubling times (Table 1). On day two, 50 µl of berbamine-containing experimental culture medium was added to achieve varying berbamine concentrations and cisplatin was additionally included in the medium for resistant cell lines. On day three, medium was replaced with 200 µl of fresh serum-free DMEM/F12 containing same berbamine and cisplatin concentrations as the day before. To evaluate potential interactions with 5-ALA, half of the cell samples were additionally treated with 5-ALA of 100 µg/ml. On day four, medium was replaced with phenol red-free DMEM/F12 (ThermoFisher) supplemented with 10% FBS and 1 mM sodium pyruvate. On day five, cell viability was tested using 20 µl CellTiter-Blue® reagent (Promega, Mannheim, Germany). Resazurin, the active component of this reagent, is a blue dye that diffuses into metabolically active cells, where it is reduced by redox enzymes to the fluorescent resorufin, which was measured 2.5 h after reagent application using a microplate reader (FLUOstar OPTIMA, BMG Labtech, Ortenberg, Germany) at excitation/emission wavelengths of 560/590 nm. Data were analysed with integrated (OPTIMA v2.0, BMG Labtech) and statistical (GraphPad Prism v10.2.3) software. Background signals measured from cell-free wells were subtracted, and IC_50_ berbamine values were calculated using the formula *log(inhibitor) vs. response—Variable slope (four parameters*) of the statistical software. Final IC_50_ berbamine values for the combination treatment were calculated as the mean concentration of 5-ALA of 100 µg/ml and 0 µg/ml treatments conducted under the same experimental conditions and are listed in Table 1.

### Measurement of PpIX accumulation in relation to berbamine

**Fig. 1:**
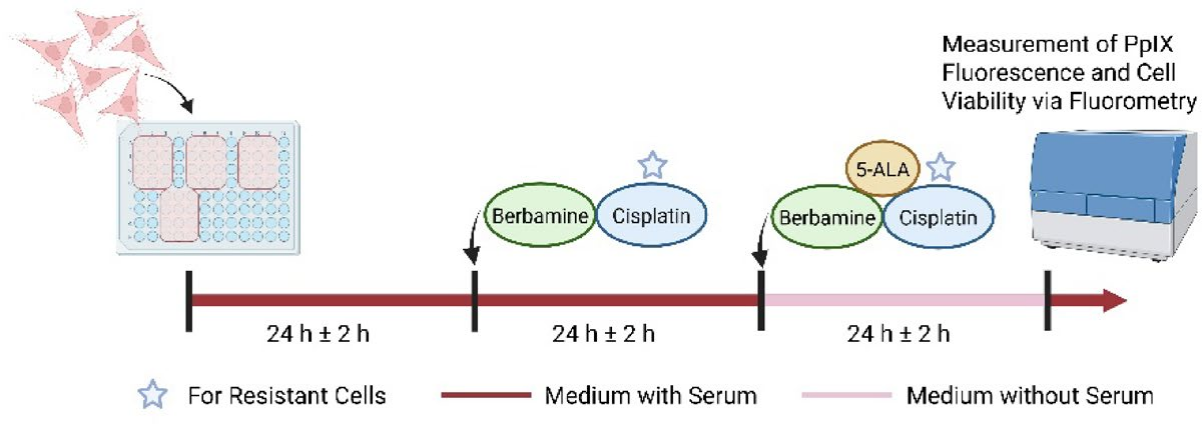
Schematic workflow of measurement of PpIX accumulation in relation to berbamine. Created in BioRender. Buchner, A. (2026) https://BioRender.com/m1j7oia.

To evaluate cellular PpIX accumulation induced by 5-ALA metabolism in the presence of berbamine, two 96-well microtiter plates were prepared identically, including triplicates for all tested concentrations of berbamine (no, ½ IC_50_ and IC_50_) and 5-ALA (0, 25, 50, and 100 µg/ml). IC_50_ berbamine values for this experiment were determined separately and are described in detail in the supplementary section (Supplementary Fig. S4). A total of 1.5 × 10^4^ cells per well were seeded in 150 µl of experimental culture medium. Plates were incubated for 24 h at 37 °C in a humidified incubator with 5% CO_2_. A schematic overview of the experimental setup is illustrated in Fig. 1. On the second day, 50 µl of fresh medium containing either ½ IC_50_ or IC_50_ berbamine concentrations or no berbamine for the control were added to each well. Cisplatin was added to the medium of cisplatin-resistant cell lines. On the third day, the medium was replaced with 200 µl of serum-free DMEM/F12 containing 5-ALA at concentrations of 0, 25, 50, or 100 µg/ml. Serum-free conditions may improve 5-ALA availability and thereby enhance its cellular uptake and metabolic conversion. Berbamine and cisplatin were added at the same concentration as on day two. On the fourth day, the medium was replaced again with 100 µl of experimental culture medium without any additives. To assess cell viability, 20 µl of CellTiter-Blue® reagent were added to the wells of half of the microtiter plates. Fluorescence intensity measurements were performed using a microplate reader (FLUOstar OPTIMA). PpIX fluorescence intensity was measured immediately (excitation/emission wavelength of 400/630 nm) after replacing the medium, while cell viability was determined after 2.5 h (excitation/emission wavelength of 560/590 nm). Raw data were analysed using integrated (OPTIMA v2.0) and statistical (GraphPad Prism v10.2.3) software. A ratio of PpIX fluorescence intensity to cell viability was calculated for all 5-ALA and berbamine concentrations by dividing the mean PpIX fluorescence intensity of each triplicate by the corresponding mean cell viability fluorescence intensity of the paired triplicate. Prior to this, background fluorescence from cell-free wells was subtracted from the corresponding wells containing cells.

### Combination therapy approach with 5-ALA-PDT and berbamine

**Fig. 2:**
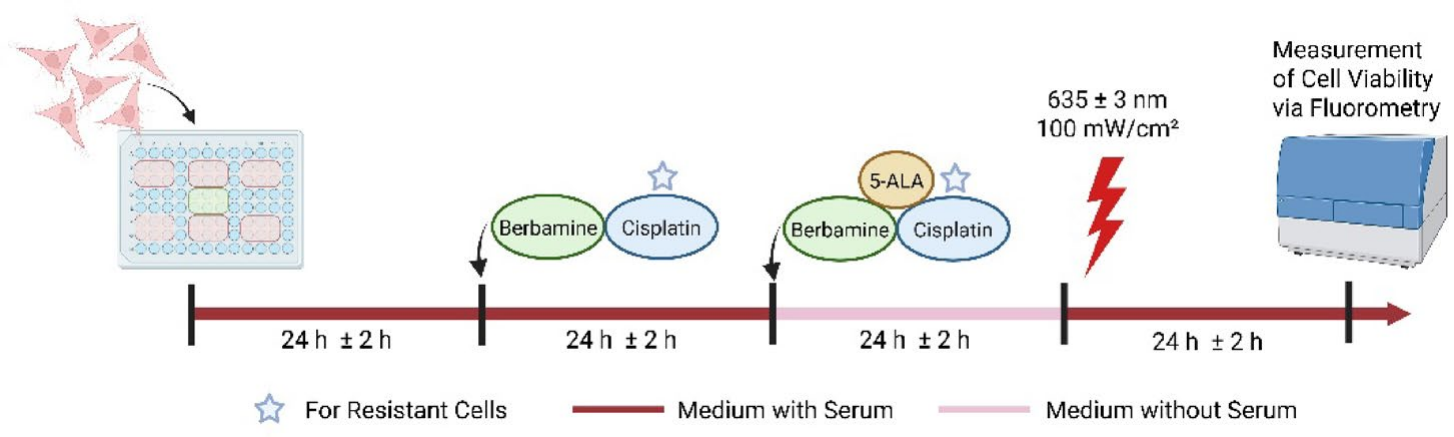
Schematic workflow of combination therapy approach with 5-ALA-PDT and berbamine. Created in BioRender. Buchner, A. (2026) https://BioRender.com/pnlojx3.

To investigate the effect of a combination treatment with 5-ALA-PDT and berbamine in the bladder cancer cell lines, cells were seeded according to their respective doubling times (Table 1). Specifically, J82, J82 LTT, J82 CSC, and RT112 CSC were seeded at 15 × 10^3^ cells per well, RT112 at 7.5 × 10^3^ cells per well, and the slow-proliferating RT112 LTT at 22.5 × 10^3^ cells per well. Cells were seeded in 96-well microtiter plates in six replicates using 150 µl of experimental culture medium per well. Plates were incubated for 24 h at 37 °C in a humidified atmosphere with 5% CO_2_. The experimental workflow is illustrated schematically in Fig. 2 for a better visualisation. On the second day, 50 µl of experimental culture medium containing berbamine were added to the wells to achieve final concentrations of ¼ IC_50_, ½ IC_50_, and IC_50_ (Table 1), as well as samples without berbamine. Control sample wells were also prepared, so that three different control groups were included: Irradiated control samples without 5-ALA, but all berbamine concentrations (C1.1–C1.4), control samples without irradiation or 5-ALA, but all berbamine concentrations (C2.1–C2.4), and control samples without irradiation or berbamine, but a 5-ALA concentration of 100 µg/ml (C3.1). Cisplatin was added to the medium of RT112 LTT and J82 LTT at previously defined concentrations. After 24 h of incubation on day three, the medium was replaced with 200 µl of serum-free DMEM/F12 containing freshly prepared 5-ALA at final concentrations of 0, 25, 50, or 100 µg/ml. Serum-free conditions were applied to minimize serum-derived interference and to improve the bioavailability and intracellular conversion of 5-ALA. Berbamine and cisplatin were added to achieve the same concentrations as on day two. On the fourth day and after 24 h of incubation, the medium was replaced with 100 µl of phenol red-free DMEM/F12 supplemented with 10% FBS and 1 mM sodium pyruvate. Cell-free wells were filled with the same medium. Cells were subsequently irradiated with red light of the wavelength of 635 ± 3 nm emitted from a diode laser system (Ceralas®, biolitec AG, Jena, Germany) at a fluence of 100 mW/cm^2^ for 0, 20, 40, 80, 160, or 320 s, corresponding to light fluence rates of 0, 2, 4, 8, 16, and 32 J/cm^2^, respectively. Irradiation from below guaranteed homogeneous light distribution in the plane of the cells, and was performed in a custom-built chamber equipped with a temperature-controlled platform, ensuring a constant temperature of 37 °C during light exposure of the cell culture plates^43^. The control samples C2.1–C2.4 and C3.1 were not irradiated. On the fifth day, 20 µl of CellTiter-Blue® reagent were added to each well. After 2.5 h of incubation at room temperature in the dark, fluorescence intensity was measured using a microplate reader (FLUOstar OPTIMA) at excitation/emission wavelengths of 560/590 nm. Raw data were analysed using integrated software (OPTIMA v2.0) and further processed in a statistical program (GraphPad Prism v10.2.3). Background fluorescence from cell-free wells was subtracted from the corresponding experimental values. To ensure that experiments with lower variability and more consistent replicates contribute more strongly to the final result of combination treatment with 5-ALA-PDT and berbamine, both means and standard deviations were calculated as weighted values across three independent experiments. Significance testing, however, was performed using unweighted values from individual replicates, as required by statistical methods such as ANOVA. This ensures statistical validity while allowing a robust and representative visualisation of trends.

### Statistical analysis

The numerical data were expressed as means ± standard deviation (SD) or with individual values. Differences between groups were assessed by two-way ANOVA followed by two-tailed Dunnett’s post hoc analysis. A significance threshold of *p* = 0.05 was applied. All statistical analyses were performed using the statistical software (GraphPad Prism v10.2.3). Due to partially small sample size, normality testing was limited; however, the tests were applied based on visual distribution checks and variance homogeneity, as they are considered robust under such conditions.

### Statement of ethical approval

The cell lines are established human bladder cancer cell lines, and no primary human samples or patient data were used in this study; therefore, ethical approval and informed consent were not required.

### Conference presentation

The presented work is part of the inaugural thesis of Muriel Kabus at the medical faculty of LMU Munich.

## Results

### ***Cell doubling time and determination of IC***_***50***_*** berbamine***

In Table 1 the results of the determination of the cell doubling time and the cell line specific IC_50_ berbamine values are shown, serving for all further experiments and data evaluations.

### Berbamine supports PpIX accumulation

**Fig. 3:**
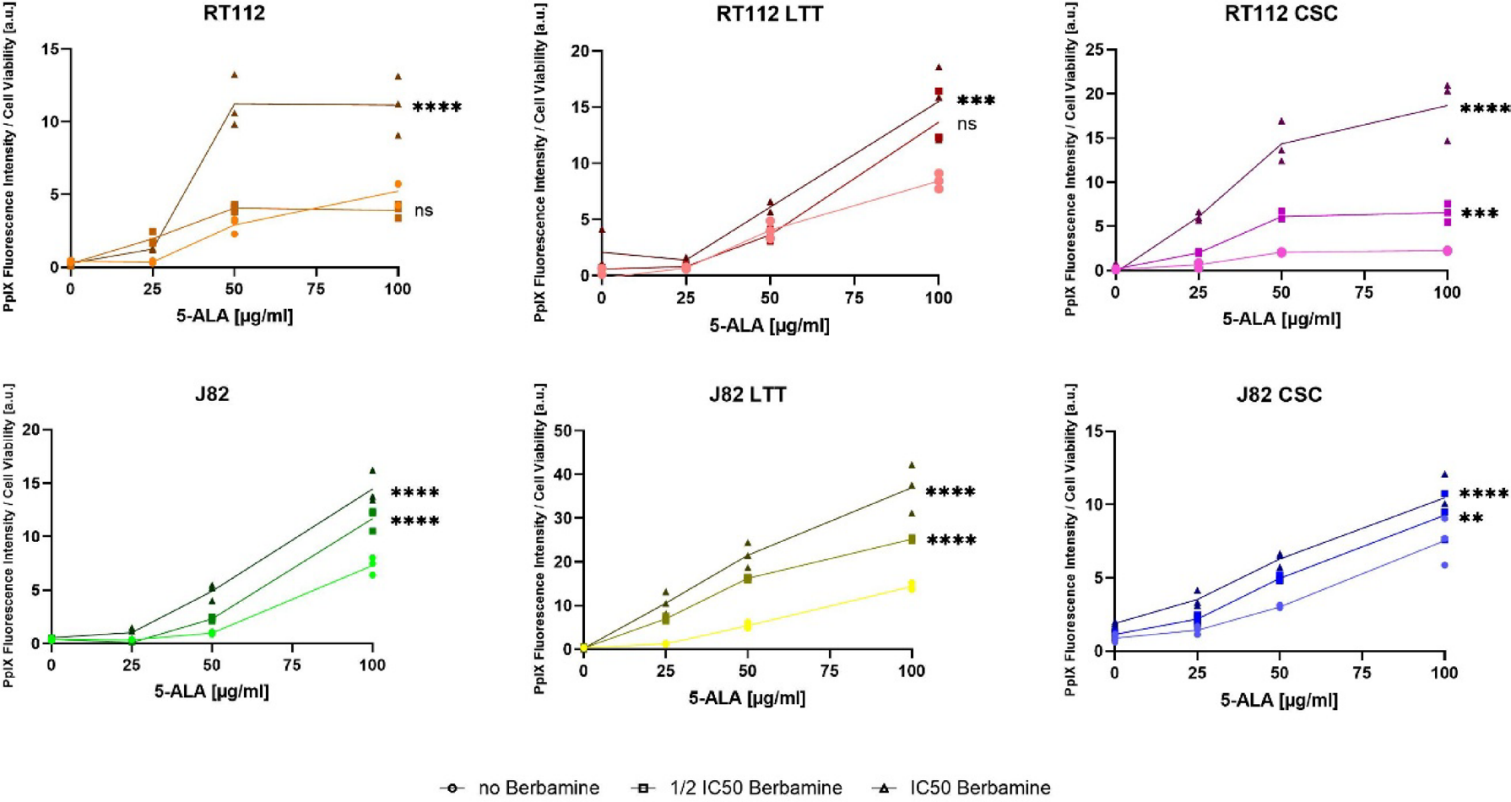
Effect of berbamine on 5-ALA-induced PpIX accumulation. RT112, RT112 LTT, RT112 CSC (top), and J82, J82 LTT, J82 CSC (bottom) were treated with 5-ALA (0–100 µg/ml) and berbamine (no, ½ IC_50_, IC_50_). PpIX fluorescence intensity was normalized to cell viability and is shown as individual values with mean as a line. Asterisks indicate significance compared to the sample without berbamine (*p < 0.05; **p < 0.01; ***p < 0.001; ****p < 0.0001).

As shown in Fig. 3, the curves with no berbamine reveal that increasing concentrations of 5-ALA resulted in higher ratios of PpIX fluorescence intensity to cell viability for all tested bladder cancer cell lines, demonstrating a clear dose-dependent increase in PpIX accumulation. A saturation effect was detected in RT112 CSC beyond 5-ALA concentrations of 50 µg/ml.

Furthermore, the IC_50_ berbamine curves demonstrate that berbamine co-treatment significantly (*p* < 0.0001, for RT112 LTT *p* < 0.001) enhanced PpIX accumulation in all tested cell lines. The strongest increase was observed in RT112 CSC treated with 5-ALA of 100 µg/ml, where the ratio of PpIX fluorescence intensity to cell viability rose more than eightfold compared to 5-ALA alone. RT112 and J82 LTT also showed a more than twofold increase under the same conditions. Only RT112 and RT112 LTT exhibited no substantial increases at ½ IC_50_ berbamine compared to the berbamine-free control. Moreover, ½ IC_50_ and IC_50_ berbamine co-treatment resulted in plateau effects in RT112 and RT112 CSC and slight flattening of the curve in J82 LTT, indicating varying degrees of PpIX accumulation saturation at a 5-ALA concentration of 50 µg/ml. When comparing maximum ratios of PpIX fluorescence intensity to cell viability at a 5-ALA concentration of 100 µg/ml and IC_50_ berbamine, values increased in the following order: J82 CSC (10.5), RT112 (11.1), J82 (14.5), RT112 LTT (17.2), RT112 CSC (18.7), and J82 LTT (37.0), highlighting cell line-specific differences in PpIX accumulation in response to combination treatment.

### Berbamine monotreatment showed antitumoral effects

**Fig. 4:**
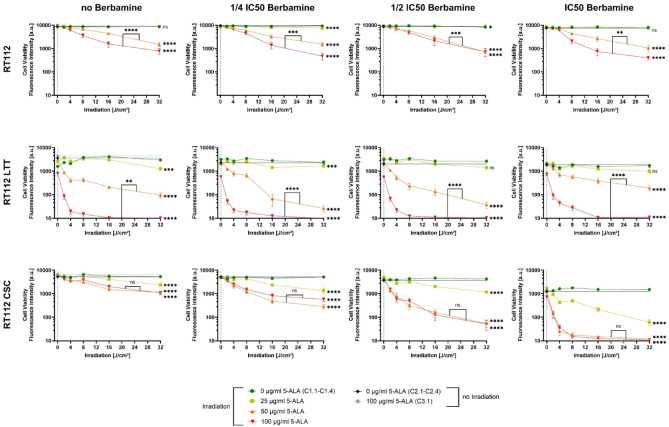
Cell viability of RT112, RT112 LTT and RT112 CSC following 5-ALA-PDT treatment with increasing irradiation doses and varying concentrations of 5-ALA and berbamine. Bars represent weighted means ± SD from three independent experiments (*n* = 3 × 6; controls without irradiation *n* = 3 × 36). Asterisks indicate significance of unweighted means compared to the irradiated control sample without 5-ALA C1.1-C1.4 (*p < 0.05; **p < 0.01; ***p < 0.001; ****p < 0.0001).

**Fig. 5:**
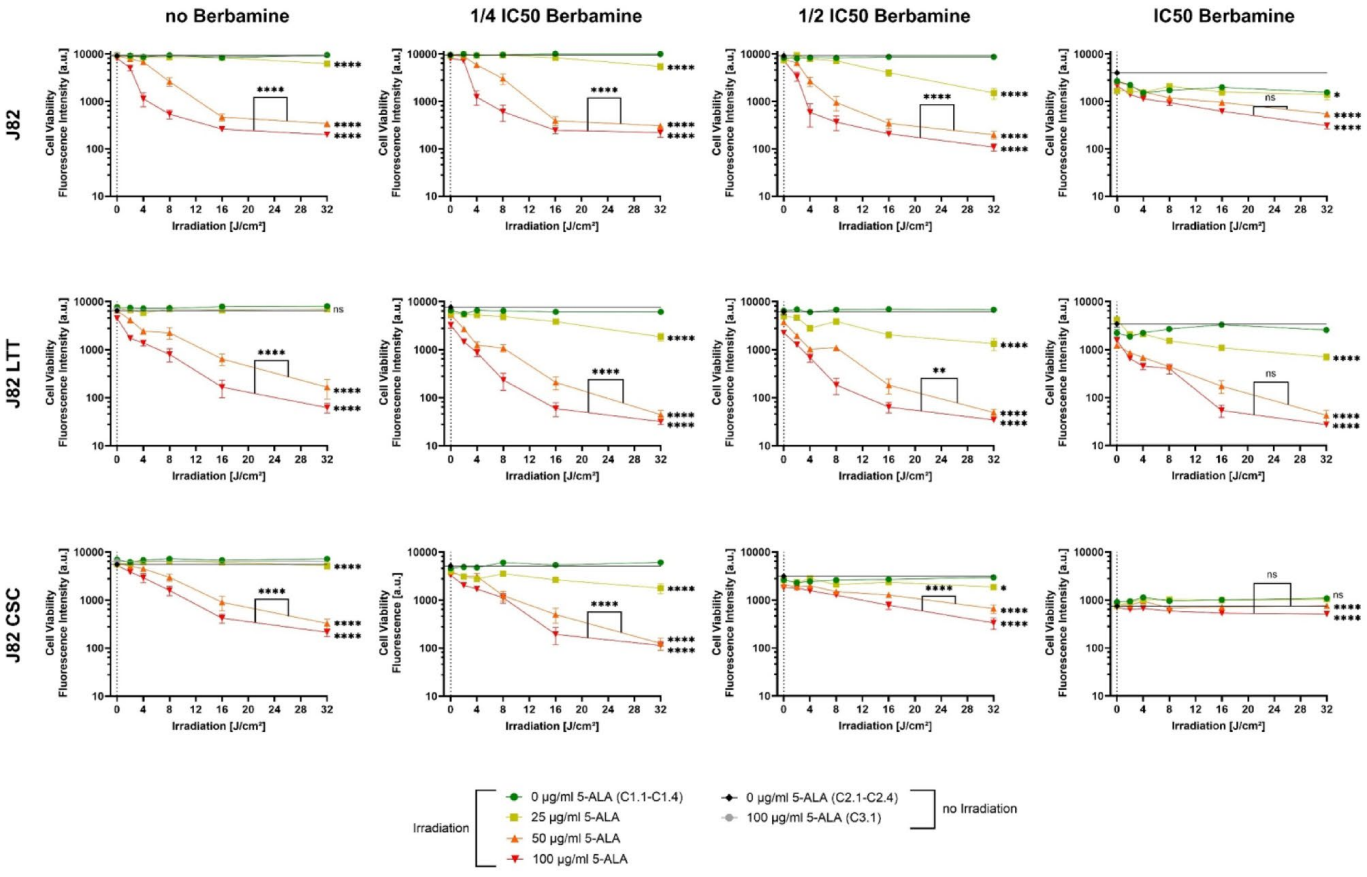
Cell viability of J82, J82 LTT and J82 CSC following 5-ALA-PDT treatment with increasing irradiation doses and varying concentrations of 5-ALA and berbamine. Bars represent weighted means ± SD from three independent experiments (*n* = 3 × 6; controls without irradiation *n* = 3 × 36). Asterisks indicate significance of unweighted means compared to the irradiated control sample without 5-ALA C1.1-C1.4 (*p < 0.05; **p < 0.01; ***p < 0.001; ****p < 0.0001).

Consistent with the cell viability assay results, berbamine monotreatment (that is treatment without the influence of 5-ALA or irradiation as part of the PDT) decreased cell viability in all six cell lines. The extent of this reduction varied between cell lines (C2.1-C2.4 of Fig. 4, RT112 variants and Fig. 5, J82 variants). Relative cell viability loss for non-irradiated 5-ALA-free controls at IC_50_ berbamine (C2.4.) compared to the ones without any berbamine (C2.1) followed the order: RT112 (23%), J82 LTT (31%), J82 (43%), RT112 LTT (45%), RT112 CSC (54%), and J82 CSC (86%). These results might suggest that the applied IC_50_ berbamine concentrations could have been underestimated for RT112 and J82 LTT, and overestimated for J82 CSC. A relative cell viability loss of 47% at ½ IC_50_ berbamine could reflect the actual IC_50_ for this cell line.

### 5-ALA-PDT monotreatment showed antitumoral effects

In all bladder cancer cell lines, 5-ALA-PDT monotreatment (that is treatment without the influence of berbamine) considerably reduced cell viability in an irradiation dose-dependent manner (Fig. 4, RT112 variants; Fig. 5, J82 variants). Irradiated samples treated with 5-ALA of 50 or 100 µg/ml showed highly significant (*p* < 0.0001) reductions in cell viability compared to the irradiated 5-ALA-free controls, decreasing progressively with increasing light doses from 0 to 32 J/cm^2^. The extent of cell viability loss due to increasing irradiation, however, varied between cell lines. Twenty-four hours after 5-ALA-PDT under maximum conditions (5-ALA of 100 µg/ml, 32 J/cm^2^), RT112 and RT112 CSC maintained residual cell viability fluorescence intensities of approximately 1000 a.u., while RT112 LTT displayed near-complete loss of cell viability, approaching the signal of cell-free wells to 10 a.u. J82 cell variants yielded fluorescence intensities between approximately 200 and 350 a.u. for J82 and J82 CSC and around 100 a.u. for J82 LTT, indicating low, but detectable, residual cell viability. Notably, RT112 CSC showed no significant difference in cell viability between 5-ALA concentrations of 50 and 100 µg/ml, suggesting PpIX saturation at 50 µg/ml. 5-ALA-PDT at 5-ALA concentrations of 25 µg/ml had no effect on RT112 and J82 LTT cells, while significant cell viability loss were detected in RT112 LTT cells (*p* < 0.001) as well as in RT112 CSC, J82 and J82 CSC cells (*p* < 0.0001).

### Combination therapy approach with 5-ALA-PDT and berbamine shows cell line-dependently altered cell viability for RT112 cell line variants

In RT112 cells, treated with 5-ALA of 100 µg/ml and irradiated, a berbamine concentration of ¼ IC_50_ already led to a noticeable reduction in cell viability compared to the berbamine-free equivalent (Fig. 4, RT112 graphs). At IC_50_ berbamine, cell viability declined more sigmoidally with increasing irradiation, deviating from the typical logarithmic pattern observed under lower concentrations of berbamine. The difference in unweighted means of cell viability between 5-ALA-PDT treatments with 5-ALA of 50 and 100 µg/ml became smaller with more berbamine (from *p* < 0.0001 to *p* < 0.01), suggesting that the cell viability at a 5-ALA concentration of 50 µg/ml also begins to drop substantially at higher berbamine concentrations and reaches cell viability rates of irradiated samples with higher 5-ALA concentrations. At the lowest 5-ALA concentration of 25 µg/ml, a highly significant reduction (*p* < 0.0001) in cell viability was detected in irradiated samples treated with ¼ IC_50_ berbamine. However, at berbamine concentrations of ½ IC_50_ and more, the additional cytotoxic effect diminished, with little (*p* < 0.05) to no significant difference compared to the 5-ALA-free irradiated control C1.3 and C1.4.

RT112 LTT cells exhibited no improved reduction in cell viability when higher concentrations of berbamine were applied (Fig. 4, RT112 LTT graphs). Specifically, for samples with 5-ALA of 25 µg/ml, a statistically significant decrease (*p* < 0.001) in cell viability was first noted compared to the irradiated control without 5-ALA of up to ¼ IC_50_ berbamine (C1.1 and C1.2). At higher berbamine concentrations, no significant differences in unweighted mean viabilities were detected anymore. Irradiated samples treated with 5-ALA of 50 µg/ml showed the lowest cell viability loss at the maximum applied light dose of 32 J/cm^2^ when combined with the full IC_50_ berbamine. Similarly, at 5-ALA of 100 µg/ml, the decline in cell viability with increasing irradiation doses at IC_50_ berbamine was slower than was observed with lower berbamine concentrations. Still, cell viability approached zero at 16 J/cm^2^, thereby confirming the full efficacy of 5-ALA-PDT at 100 µg/ml under these conditions.

Compared to all tested cell lines, the cancer stem cell line RT112 CSC exhibited the strongest increase in sensitivity to 5-ALA-PDT when berbamine concentration was elevated (Fig. 4, RT112 CSC graphs). Notably, no significant differences in cell viability were noted between irradiated samples treated with 5-ALA of 50 µg/ml and 100 µg/ml across all berbamine concentrations, suggesting that 50 µg/ml was sufficient to reach PpIX saturation. At these saturating 5-ALA levels, increasing berbamine concentrations led to a stronger decrease in cell viability with rising irradiation doses. To highlight the strength of the combination effect, it can be emphasised that at a 5-ALA concentration of 25 µg/ml and IC_50_ berbamine, cell viability reached levels even lower than those detected at 5-ALA concentrations of 50 and 100 µg/ml without berbamine.

### Combination therapy approach with 5-ALA-PDT and berbamine shows concentration-dependently diminished cell viability for J82 cell line variants

In J82 cells, an initial concentration-dependent reduction in cell viability during combination treatment was observed when berbamine concentrations increased from 0 to ¼ IC_50_ and ½ IC_50_ across all 5-ALA concentrations (Fig. 5, J82 graphs). Interestingly, at IC_50_ berbamine, the cytotoxicity induced by 5-ALA-PDT was reduced, although cell viability had already declined 43% due to berbamine. Most notably, at a 5-ALA concentration of 25 µg/ml and IC_50_ berbamine, unweighted mean cell viability was only marginally lower than in the irradiated 5-ALA-free control C1.4 (*p* < 0.05). In irradiated samples treated with 5-ALA of 50 or 100 µg/ml, cell viability at IC_50_ berbamine remained more than twice as high as at ½ IC_50_ berbamine under maximum irradiation.

The cisplatin-resistant J82 LTT cells showed a consistent decline in cell viability with increasing berbamine concentrations (Fig. 5, J82 LTT graphs). Without berbamine influence, the effects of 5-ALA-PDT with 5-ALA of 25 µg/ml did not significantly differ from the irradiated 5-ALA-free control C1.1. However, starting from ¼ IC_50_ berbamine, cell viability decreased significantly (*p* < 0.0001) with increasing light dose. At higher berbamine concentrations, irradiated samples with 5-ALA of 50 µg/ml aligned with the ones with 5-ALA of 100 µg/ml in falling below a cell viability fluorescence intensity of 100 a.u., indicating near-complete loss of viable cells.

The attenuated, weaker decrease in cell viability with increasing irradiation doses observed in J82 cells treated at IC_50_ berbamine was even more pronounced in J82 CSC (Fig. 5, J82 CSC graphs). At ¼ IC_50_ berbamine, 5-ALA-PDT led to substantial cell viability loss across all 5-ALA concentrations. However, at ½ IC_50_, this effect is diminished, and the cell viability under maximal treatment conditions (5-ALA of 100 µg/ml, 32 J/cm^2^) remained higher than in the berbamine-free equivalents, despite the initial cytotoxic effect of berbamine monotreatment. At IC_50_ berbamine concentration, the cell viability curves flattened further, and unweighted mean viabilities of samples with 5-ALA of 25 µg/ml showed no significant difference compared to the 5-ALA-free control C1.4.

## Discussion

### Berbamine is a cytotoxic agent in bladder cancer

**Fig. 6:**
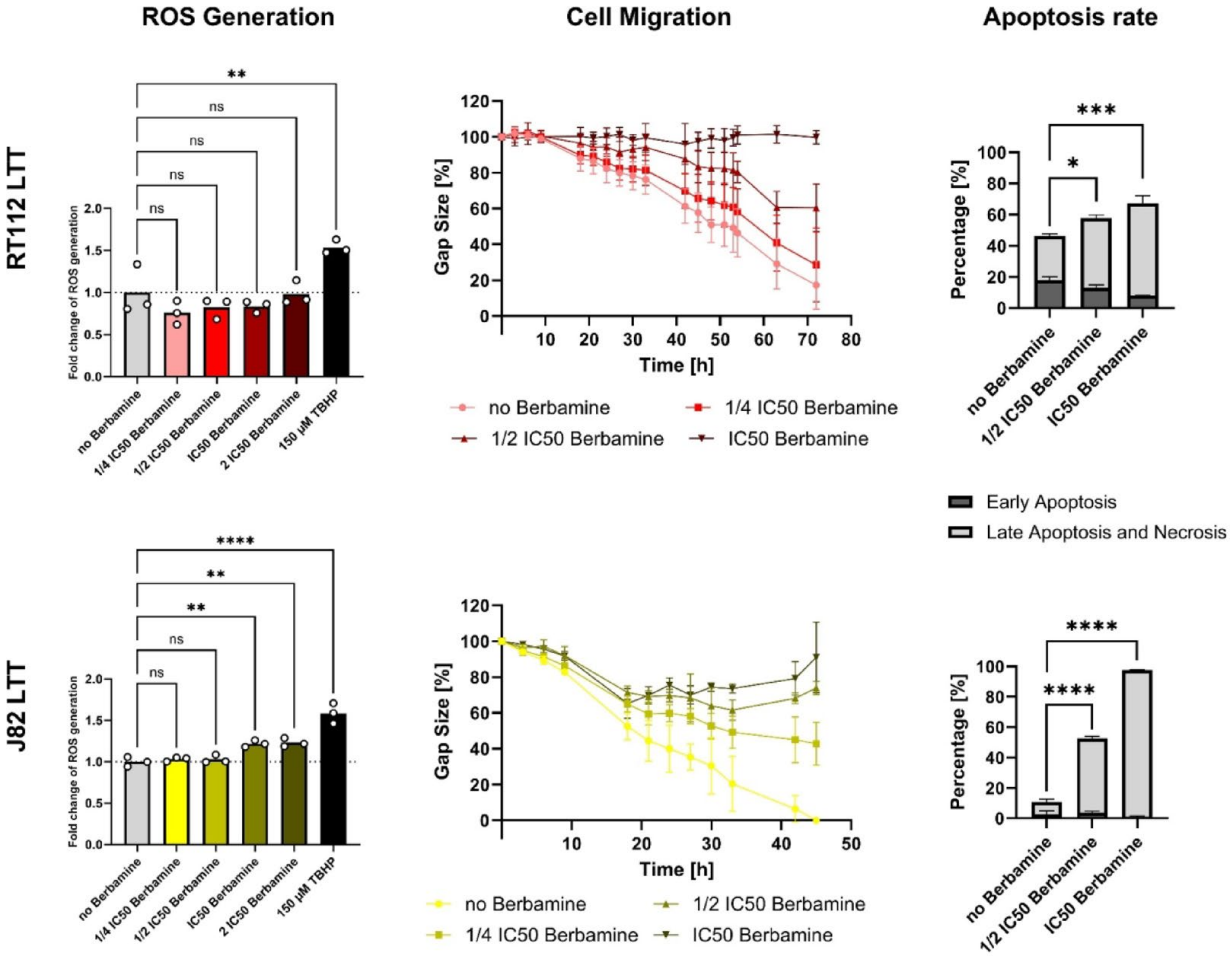
Berbamine has cytotoxic effects in ROS generation, cell migration and apoptosis rate for cisplatin-resistant cell lines RT112 LTT and J82 LTT. Tert-butyl hydroperoxide (TBHP; 150 µM) served as positive control for determination of reactive oxygen species (ROS). Detailed data presentation for all cell lines are available in the supplementary section (Supplementary Figs. S6, S9 and S10). Data represent mean ± SD (*n* = 3). Asterisks indicate significance relative to the control without berbamine (*p < 0.05; **p < 0.01; ***p < 0.001; ****p < 0.0001).

The present study demonstrates that berbamine exerts antitumoral effects as an in vitro monotreatment in the tested variants of the bladder cancer cell lines J82 and RT112. Berbamine also reduced cell viability in the combination treatment experiments under irradiation via 5-ALA-PDT (Table 1, C2.1–C2.4 of Figs. 4, 5). Additional supplementary investigations confirmed its cytotoxic properties by showing decreased cell migration and invasion as well as increased apoptosis and necrosis rates (Supplementary Figs. S6, S7, S8, S9). Berbamine did not significantly increase intracellular ROS production within the concentration range up to the IC_50_ berbamine (Supplementary Fig. S10). A notable exception was observed in J82 LTT cells, where ROS production was markedly elevated already at the IC_50_ berbamine concentration (Fig. 6, ROS generation of J82 LTT at IC_50_ berbamine). It had been reported that in in vitro experiments performed with the bladder cancer cell lines 5637 and T24, higher exposures to berbamine as IC_50_ stimulated ROS generation within 24 h in downregulating key antioxidant proteins such as Nrf2, HO-1, SOD2, and GPx-1^37^. Therefore, additional mechanisms should be discussed or kept in mind in context of this paper to explain the antitumoral effects of berbamine at the tested concentration ranges.

Berbamine has been shown to inhibit the ROS-sensitive NF-κB signalling pathway in several malignancies, avoiding chronic inflammation, tumour progression, therapy resistance, and epithelial-mesenchymal transition, which is crucial for cell migration and invasion^37,44^. It has been reported that berbamine is able to stop tumour growth and support apoptosis in various cancer entities, for instance, in inhibiting CAMKII^45,46^. Furthermore, in bladder cancer, increasing the Bax/Bcl-2 ratio and inducing an S-phase cell cycle arrest through modulation of key checkpoint regulatory proteins could also be identified as a mechanism that inhibits tumour growth^37^.

The cisplatin-resistant cell lines RT112 LTT and J82 LTT exhibited the lowest IC_50_ berbamine values in combination treatment experiments (Table 1). Also, they showed stronger reductions in cell migration and greater increases in apoptosis and necrosis compared to their cisplatin-sensitive counterparts (Fig. 6, cell migration and apoptosis rate of RT112 LTT and J82 LTT; Supplementary Fig. S6, cell migration of all cell lines; Supplementary Fig. S9, apoptosis rate of all cell lines). Berbamine has been shown to enhance the antitumoral effects of chemotherapeutic agents such as cabazitaxel in prostate cancer by inhibiting IGF2BP1 and STAT3^47^, as well as to potentiate the efficacy of sorafenib in hepatocellular carcinoma^48^ and gefitinib in pancreatic cancer cells through STAT3 inhibition^49^. To date, however, no studies have specifically addressed whether berbamine enhances cisplatin sensitivity, particularly in bladder cancer. The presented findings thus provide initial evidence that berbamine may act as a promising adjuvant to cisplatin-based chemotherapy, particularly in drug-resistant tumour cell populations.

The cancer stem cells RT112 CSC and J82 CSC exhibited the highest IC_50_ berbamine values for the combination treatment experiments (Table 1), suggesting a greater tolerance to berbamine compared to their parental counterparts. In supplementary experiments, their invasive capacity was less effectively reduced than that of the corresponding RT112 and J82 cell lines (Supplementary Fig. S7). This increased tolerance may be attributed to characteristic features of CSCs, as it has been shown that the COX2/PGE2-let-7 axis and YAP1 signalling interact in promoting SOX2 expression, thereby supporting CSC enrichment and resistance to chemotherapy^50^. Transcriptional regulators such as SOX4 and OCT4 have been identified as key players in sustaining bladder CSC properties^51,52^. Additionally, ABCG2 contributes to CSC-mediated resistance to chemotherapy and radiotherapy by facilitating the efflux of, e.g., cytotoxic agents^16^. Supplementary analyses of migration and apoptosis (Supplementary Figs. S6, S9), however, revealed equal or greater berbamine sensitivity in CSCs compared to parental cell lines, indicating enhanced effects of berbamine on these properties in CSCs.

### 5-ALA-PDT reduces cell viability and generates more ROS through berbamine-accelerated PpIX accumulation in bladder cancer cells

Previously reported antitumoral effects of 5-ALA-PDT as a monotreatment without berbamine in bladder cancer cell lines^34^ can be confirmed by the presented experiments. Increasing irradiation doses with 635 ± 3 nm laser light of up to 32 J/cm^2^, as well as increasing 5-ALA concentrations up to 100 µg/ml, generally resulted in greater reductions in cell viability (graphs with no berbamine of Figs. 4, 5). Differences in residual cell viability under maximum 5-ALA-PDT conditions of 5-ALA of 100 µg/ml and irradiation of 32 J/cm^2^ between the cell lines may, in part, be attributable to their varying doubling times (Table 1), as viable cells can continue to proliferate during the 24-h period following irradiation until cell viability test.

The findings are consistent with previous studies^30^, demonstrating that 5-ALA administration leads to intracellular accumulation of PpIX in bladder cancer cells (Fig. 3). PpIX accumulation increased with higher 5-ALA concentrations, reaching partial saturation in RT112 and RT112 CSCs, and resulted in enhanced ROS generation across all tested cell lines (Supplementary Fig. S11). ROS production also rose with increasing irradiation. CSCs exhibited comparable responses in 5-ALA-PDT sensitivity to their parental cell lines. In contrast to what has been reported for head and neck CSCs^23^ and glioma CSCs^24^, this finding indicates that bladder cancer CSCs do not necessarily exhibit increased sensitivity to 5-ALA-PDT. The therapeutic efficacy may depend on tumour type or may require combination with supportive agents such as berbamine, as demonstrated in this study.

On top of the individual cytotoxic effects of 5-ALA-PDT and berbamine adding up in co-treated, irradiated samples, a reinforcing effect of the combined treatment beyond simple addition was detected as well. The extent and pattern of this interaction varied between the different cell types.

J82 LTT showed a simple correlation of increasing loss of cell viability and higher concentrations of berbamine (Fig. 5). Low 5-ALA concentrations of 25 µg/ml did not significantly reduce cell viability, whereas the addition of berbamine led to a highly significant decrease (*p* < 0.0001). These findings are in line with the highly increased PpIX accumulation (Fig. 3) and higher ROS generation due to either berbamine or 5-ALA-PDT (Supplementary Figs. S10, S11). Slight saturation effects in PpIX accumulation of J82 LTT beginning at 5-ALA concentrations of 50 µg/ml (Fig. 3) may explain the gradual convergence of samples treated with 5-ALA of 50 and 100 µg/ml at higher berbamine concentrations in combination treatment (Fig. 5). These investigations suggest that both 5-ALA-PDT and berbamine can enhance the cytotoxic effects of cisplatin in cisplatin-resistant bladder cancer cell lines. The results align with previous studies in various solid tumours, where 5-ALA-PDT has been shown to increase cisplatin efficacy through mechanisms such as enhanced ROS generation and apoptosis induction^52–54^. In J82 and J82 CSC cells, a seemingly nonlinear concentration-dependent effect of berbamine was observed, with the greatest reduction in cell viability at ½ IC_50_ berbamine in J82 and at ¼ IC_50_ in J82 CSC (Fig. 5). These findings suggest that the enhancing effect of 5-ALA-PDT and berbamine co-treatment may reach an optimal concentration range in these cell lines, beyond which higher berbamine concentrations may mask or attenuate further 5-ALA-PDT-induced cytotoxicity. Interestingly, PpIX accumulation and ROS generation showed an approximately linear increase with rising berbamine concentrations. It can be hypothesized that cells surviving the selective pressure of berbamine upregulate cellular defence mechanisms, thereby becoming more resistant to subsequent 5-ALA-PDT treatment and ROS exposure. To elucidate the mechanisms underlying the concentration-dependent effects of berbamine during laser irradiation, further extensive in vitro studies, like assessing ROS generation during co-treatment with 5-ALA-PDT and berbamine, are warranted. In future, it remains to be precisely investigated whether J82 LTT cells and RT112 cell line variants would exhibit similar cell viability responses at higher berbamine concentrations in combination treatment. At the IC_50_ berbamine concentration, such effects have not yet been clearly observed.

The efficacy of combination treatment in RT112 cells was dependent on the concentration of 5-ALA. Increasing berbamine concentrations initially enhanced but subsequently attenuated cell viability loss at 5-ALA concentrations of 25 µg/ml, whereas higher 5-ALA concentrations were associated with a more steady but only slight increase in cell viability loss under combination treatment (Fig. 4). At a 5-ALA concentration of 50 µg/ml, cell viability patterns increasingly resembled those at 100 µg/ml with rising berbamine concentrations, statistically suggesting a saturation effect. This is supported by the data of PpIX accumulation (Fig. 3), which showed saturation up to ½ IC_50_ berbamine. Combination treatment experiments in RT112 LTT cells showed no enhancement of 5-ALA-PDT efficacy by berbamine (Fig. 4). At a 5-ALA concentration of 100 µg/ml, cell viability loss remained consistently being close to zero starting at an irradiation of 16 J/cm^2^ across all berbamine concentrations, suggesting no loss in 5-ALA-PDT efficacy in this cell line but also no reinforcing interaction under these combination treatment conditions. Notably, RT112 LTT showed the lowest increase in PpIX accumulation under treatment with berbamine (Fig. 3), supporting this observation. Among all tested cell lines, RT112 CSCs showed the greatest enhancement of 5-ALA-PDT efficacy to berbamine (Fig. 4). Cell viability fluorescence intensity dropped dramatically from above 1000 a.u. to 10 a.u. under the strongest treatment conditions (32 J/cm^2^, 5-ALA of 100 µg/ml). While greater 5-ALA-PDT sensitivity was not observed in the performed experiments without berbamine, the combination with this agent clearly enhanced 5-ALA-PDT efficacy in RT112 CSCs compared to parental RT112 cells. Notably, 5-ALA of 50 µg/ml already produced the maximal reduction in cell viability, with no significant improvement at 100 µg/ml, which corresponds to the observed plateau in PpIX fluorescence intensity to cell viability across berbamine concentrations (Fig. 3). At IC_50_ berbamine, the PpIX fluorescence intensity to cell viability increased more than eightfold compared to 5-ALA alone, representing by far the highest rise observed. This supports the assumption that berbamine-induced increases in PpIX accumulation contribute to the improved efficacy of the combination treatment.

The data from combination treatment experiments suggest that berbamine could help face intrinsic resistance mechanisms, such as antioxidant defence systems^36^, thereby addressing a major limitation in current PDT strategies. In vivo evidence supports the potential of berbamine as a PDT enhancer, as recent studies have demonstrated that co-administration with zinc phthalocyanine-based PDT results in a tumour inhibition that exceeds the effect of either monotreatment alone^39^.

Comparing the J82 and RT112 cell line variants revealed distinct response profiles. Stronger berbamine-induced apoptosis rates in J82 cell line variants (Supplementary Fig. S9) may explain the greater cell viability loss in J82 LTT with higher berbamine concentrations compared to RT112 LTT under combination treatment, despite requiring lower cisplatin concentrations. Additionally, J82 and J82 CSCs indicated nonlinear responses to increasing berbamine concentrations during 5-ALA-PDT treatment, suggesting mechanisms beyond simple concentration-dependent cytotoxicity. It remains to be elucidated whether RT112 variants would manifest analogous responses if berbamine induced apoptosis to a comparable extent. Future experiments will address this by testing higher berbamine concentrations in RT112 cell line variants to evaluate potential convergence of treatment responses.

## Limitations

As all experiments were conducted exclusively in vitro*,* therefore, they may not fully reflect the complexity of the tumour environment in vivo. Using a single cell type in two-dimensional culture does not replicate the structural and cellular heterogeneity found in native tissue. Moreover, the artificial conditions, including the exposure to FBS, which urothelial cells do not encounter under physiological circumstances, further limit the model. To increase translational value, future studies should employ more advanced and physiologically relevant models that better replicate the native tissue environment, like complex three-dimensional bladder scaffolds^55,56^. In addition, cells from healthy, non-tumorous tissue were not included as a control, which precludes conclusions about the potential adverse effects of the combination treatment. The reproducibility of results can vary due to researcher-specific laboratory techniques and laboratory-specific conditions for cell culture, such as light and temperature. These factors can influence the cell cycle and doubling time. Furthermore, although the findings point towards the involvement of several cell signalling pathways and cellular interactions, the precise molecular mechanisms underlying the effects of berbamine were not directly investigated. Thus, further in vivo studies and mechanistic analyses will be essential to validate the observed effects and their explanations herein, and to explore potential enhancing pathways in greater detail. Whether the discovered reinforcing effect on the efficacy of 5-ALA-PDT by berbamine relies on synergistic effects requires further experiments and statistical analysis.

## Conclusion and future outlook

Berbamine could be a promising new addition to 5-ALA-mediated photodynamic therapy, not only due to its properties of enhancing PpIX accumulation, but also for its selective effects on 5-ALA-PDT and berbamine co-treatment across different cell lines. These findings suggest that the therapeutic potential of berbamine as an enhancer for 5-ALA-PDT efficacy is not only concentration-dependent but also highly influenced by the biological characteristics of each cell line, including cisplatin resistance and cancer stem cell properties. By not yet fully-discovered interaction of intrinsic resistance mechanisms in cancer cells, berbamine could contribute to improve the overall outcome of clinical applications of 5-ALA-PDT.

Future in vivo research should focus on translating these results into preclinical and clinical applications to improve treatment efficacy in therapy-resistant cases and lower disease-associated mortality. As more research should focus on combating the resilient CSCs with their complex resistance mechanisms in bladder cancer^17^, the findings suggest that the 5-ALA-PDT and berbamine co-treatment shows great potential to overcome this challenge. Considering that 5-ALA-PDT is a cost-effective modality and that both 5-ALA and berbamine are available at low cost in the required concentrations, implementing this combination approach may also help reduce overall treatment budgets.

Individually testing of bladder cancer biopsy material to be sensitive to 5-ALA-PDT and berbamine co-treatment might be a necessary step before starting combination therapies in patients. This could be done simultaneously with molecular and genomic testing, which is already standard for advanced or metastatic urothelial carcinoma^4^, to guide clinical decision-making and minimise delays in initiating subsequent therapies.

## Supplementary Information

Below is the link to the electronic supplementary material.

Supplementary Material 1

## Data Availability

The datasets generated during and/or analysed during the current study are available from the corresponding author on reasonable request.
