# Supplementary material for: Combining 5-ALA-PDT with berbamine as an in vitro multimodal therapy approach against bladder cancer cells
Source: Sci Rep. 2026 Apr 1;16:11228. doi: 10.1038/s41598-026-46092-x (PMC13046741; doi:10.1038/s41598-026-46092-x)
Supplement: Supplementary file 1 — Supplementary Material 1 [file 41598_2026_46092_MOESM1_ESM.pdf]

Supplements

Combining 5-ALA-PDT with berbamine as an in vitro multimodal therapy approach against bladder cancer cells

Muriel Kabus[a,b,*,1], Maximilian Aumiller[b,c], Adrain Rühm[b,c], Thomas Pongratz[b,c], Michèle J. Hoffmann[d], Alexander Buchner[c], Ronald Sroka[b,c] and Heike Pohla[a,c]

[a] *Laboratory of Tumor Immunology, LIFE Center, LMU University Hospital, LMU Munich, Fraunhoferstr. 20, Planegg 82152, Germany*
[b] *Laser Research Laboratory, LIFE Center, LMU University Hospital, LMU Munich, Fraunhoferstr. 20, Planegg 82152, Germany*
[c] *Department of Urology, LMU University Hospital, LMU Munich, Marchioninistr. 15, Munich 81377, Germany*
[d] *Department of Urology, Medical Faculty and University Hospital Duesseldorf, Heinrich Heine University Duesseldorf, Moorenstr. 5, 40225 Duesseldorf, Germany*
[*] *Corresponding author. Email address: Muriel.Kabus@campus.lmu.de*
[1] *The presented work is part of the inaugural thesis of Muriel Kabus at the medical faculty of LMU Munich*

1. Cell culture, general cell handling procedures, and sphere formation

1.1. General cell handling procedures

For all experiments, adherent cell lines were harvested at 90-95% confluency using 1% trypsin/EDTA (ThermoFisher, Carlsbad, CA, USA), stained 1:1 with 0.4% trypan blue (ThermoFisher), and counted under a light microscope using a Neubauer haemocytometer (Labor Optik, Lancing, UK). Cells were then seeded at defined densities according to the respective experimental requirements. All cell lines were maintained and incubated at 37 °C in a humidified incubator with 5% CO_2. To minimize evaporation during incubation in multiwell plates, the plates were placed in a metal container filled with sterile water, and empty wells in 96-well formats were filled with 100 µl phosphate-buffered saline (PBS; ThermoFisher).
Unless otherwise specified, cell lines were cultured in Dulbecco's Modified Eagle Medium (DMEM) GlutaMAX™ (4.5 g/l D-glucose; ThermoFisher), supplemented with 10% foetal bovine serum (FBS; Biosell, Ennigerloh, Germany) and 1 mM sodium pyruvate (ThermoFisher). This composition is hereafter referred to as experimental culture medium. For routine culturing of RT112 and J82 cells, however, Roswell Park Memorial Institute (RPMI) 1640 medium (ThermoFisher), supplemented with 10% foetal bovine serum (FBS; Biosell, Ennigerloh, Germany), 1% minimum essential medium non-essential amino acids (MEM NEAA, ThermoFisher), 1 mM sodium pyruvate, and 2 mM L-glutamine (ThermoFisher) was used. To maintain stable chemotherapy resistance, RT112 LTT and J82 LTT cells were continuously exposed to 15.5 µg/ml (51.5 µM) and 2.5 µg/ml (8.3 µM) cisplatin (Selleckchem, Houston, TX, USA), respectively, throughout all experiments.
For experiments containing berbamine (Selleckchem), a 50 mM stock solution was prepared by dissolving 1 g of berbamine powder in 29.34 ml of dimethyl sulfoxide (DMSO; WAK-Chemie, Steinbach, Germany).

1.2. Morphological and proliferative characteristics of adherent cell lines

Morphological and proliferative differences were observed among the four adherent bladder cancer cell lines. Representative phase-contrast images are presented in Supplementary Fig. S 1 and were acquired using an inverted light microscope (Leica DM IL, Leica Microsystems, Wetzlar, Germany) equipped with a USB digital camera (MikrOkular Full HD, Bresser GmbH, Rhede, Germany) and processed with its provided image acquisition software (CamLabLite™, Bresser GmbH). The most notable morphological differences were observed between cisplatin-sensitive and -resistant cell lines. RT112 and J82 cells had a compact, polygonal shape, with RT112 appearing particularly small with high optical contrast. By contrast, RT112 LTT and J82 LTT cells adopted a more elongated, spindle-shaped morphology and developed more pronounced cellular extensions. While RT112 and J82 cells grew densely and adhered uniformly to the culture surface, RT112 LTT and J82 LTT frequently showed partial detachment. In RT112 LTT cultures, the detached cells appeared dark and non-viable, which is indicative of cellular stress or death. By contrast, J82 LTT cultures showed bright, rounded cells that were still viable and had detached from the monolayer. Preliminary visual inspection already indicated

differences in proliferation dynamics among the cell lines. These observations were validated and quantified by measuring cell doubling times.

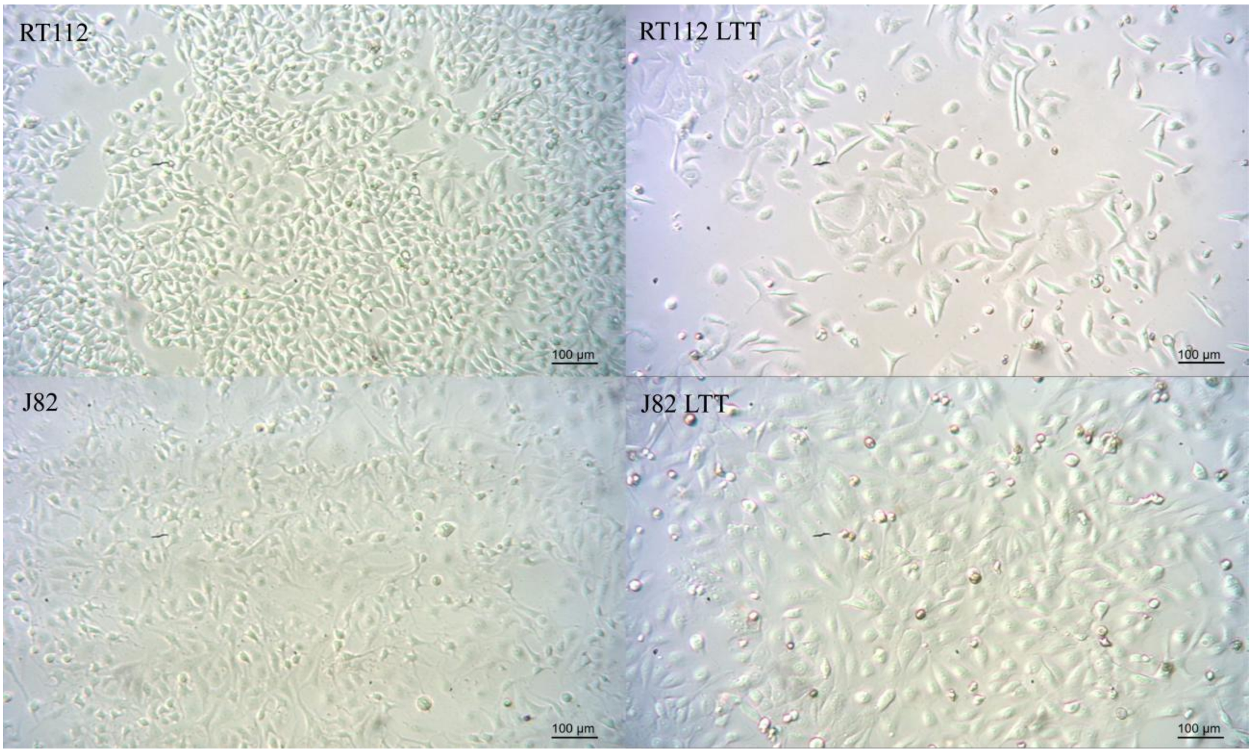

Supplementary Fig. S 1: Morphological comparison of adherent cell lines under phase-contrast microscopy. Representative images of RT112, RT112 LTT, J82 and J82 LTT 48 h after seeding 19×10^3 cells/cm². Images were acquired using a light microscope at 40× magnification.

1.3. CSC sphere formation and isolation

Cancer stem cells were generated from the cisplatin-sensitive cell lines RT112 and J82 by culturing them in DMEM and Ham's F12 (DMEM/F12; ThermoFisher) supplemented with 2% of 50× B-27 (ThermoFisher), 10 ng/ml basic fibroblast growth factor (bFGF; Sigma-Aldrich, St. Louis, MO, USA), and 10 ng/ml epidermal growth factor (EGF; Sigma-Aldrich). RT112 and J82 were first treated with 5 ml of StemCell Accutase® (ThermoFisher) and incubated for approximately 10 minutes at 37 °C in a 5% CO_2 atmosphere to detach them. Subsequently, 10^6 cells were counted and seeded into a 75 cm² ultra-low attachment flask (Corning, NY, USA) containing 10 ml of CSC medium without serum and incubated for seven days. Without the ability to adhere to the flask surface, differentiated cells undergo apoptosis, whereas CSCs survive and form spheres. Sphere formation was monitored daily at 100× magnification using an inverted light microscope (Leica DM IL) equipped with a USB digital camera (MikrOkular Full HD). The formation of RT112 CSC and J82 CSC spheres is displayed in Supplementary Fig. S 2.

After seven days of culture, the generated spheres were collected together with CSC medium and centrifuged at 355 × g for 4 minutes. After discarding the supernatant, the cell pellet was gently resuspended in 5 ml of StemCell Accutase®, followed by an incubation at 37 °C for 10 to 13 minutes in a water bath for dissociation. Prolonged incubation was avoided to prevent potential adverse effects, such as reaggregation of the cells. The resulting single-cell suspension was filtered through a 40 µm nylon cell strainer (Corning), followed by a second centrifugation step at 411 × g for 4 minutes. The supernatant was removed, and the pellet was again gently resuspended in an appropriate medium for downstream applications. Cell counting was performed using a Neubauer haemocytometer, enabling either the initiation of an experiment or a subsequent round of CSC enrichment over another seven-day period.

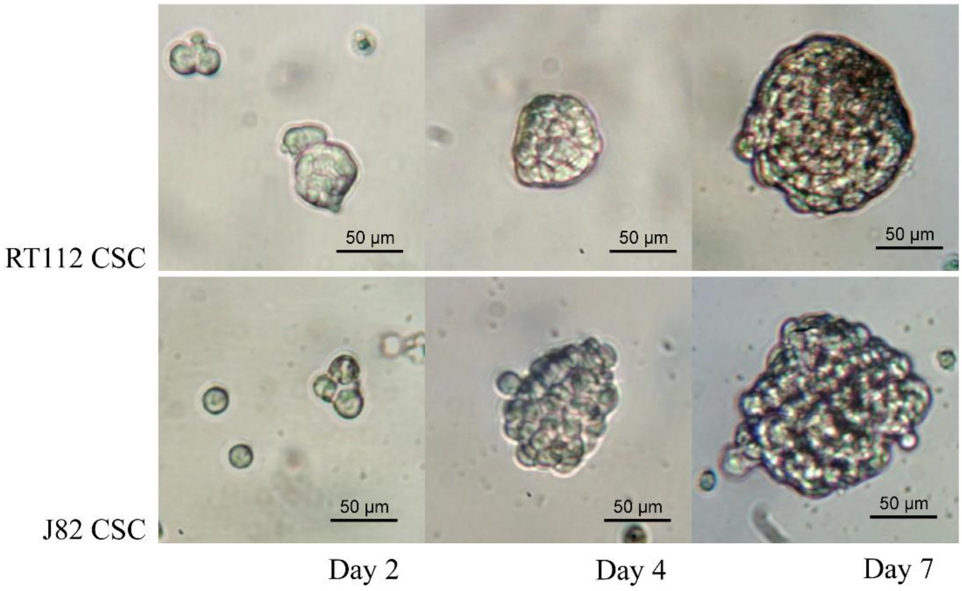

Supplementary Fig. S 2: Sphere formation of RT112 CSC and J82 CSC under phase-contrast microscopy at days 2, 4, and 7 after seeding of 1.3 × 10^4 cells/cm². Images were acquired using a light microscope at 100× magnification. RT112 CSC spheres were more compact and round-shaped than those of J82 CSC.

2. Quantification of cell doubling time

2.1. Materials and methods

To assess cell proliferation rates, 1.6 × 10^3 cells/cm² were seeded in experimental culture medium, giving a total of 4 × 10^5 cells per 25 cm² culture flask (ThermoFisher). Cisplatin was added at defined concentrations for cisplatin-resistant cell lines. Cells were harvested and counted after approximately 48 h and 96 h of incubation. Doubling times were calculated using a statistical software (GraphPad Prism v10.2.3, GraphPad Software, San Diego, CA, USA) based on an exponential (Malthusian) growth model [42], with a higher weighting assigned to the initial cell count.

2.2. Results

All cell lines demonstrated proliferative capacity over time, with varying doubling times, as illustrated in Supplementary Fig. S 3. The cisplatin-sensitive cell lines RT112 and J82 exhibited the shortest doubling times for their variant groups of over 24.0 h and 44.8 h, respectively. Intermediate doubling times were observed for the cancer stem cells RT112 CSC (31.3 h) and J82 CSC (47.3 h), as well as the cisplatin-resistant J82 LTT (45.9 h). RT112 LTT showed the slowest proliferation rate, with a doubling time of 60.1 h, likely reflecting the effects of continuous exposure to 15.5 µg/ml cisplatin. Based on these proliferation rates, seeding densities were adapted accordingly in selected experiments: halved for RT112, and increased 1.5-fold for RT112 LTT.

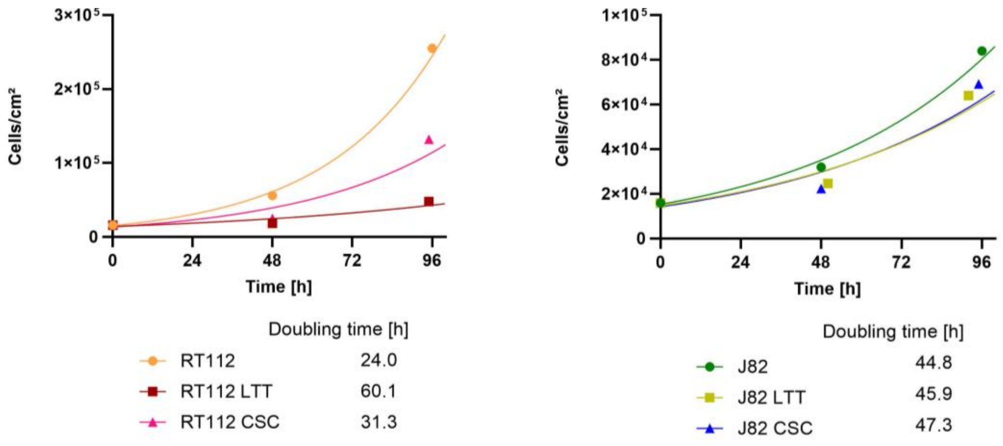

Supplementary Fig. S 3: Proliferation curves and doubling times of RT112 and J82 cell line variants. Growth kinetics of RT112, RT112 LTT, and RT112 CSC (left) as well as J82, J82 LTT, and J82 CSC (right) over 48 h and 96 h (n = 1). Doubling times [h] were calculated based on a fit according to exponential (Malthusian) growth models [42].

3. Determination of IC_{50} berbamine and cisplatin

3.1. For characterization of bladder cancer properties and measurement of PpIX accumulation

Materials and methods

To determine the half-maximal inhibitory concentration (IC_{50}) of berbamine and cisplatin for each cell line, cell viability was assessed via fluorometry. Triplicates of 5×10^3 cells per well were seeded in 80 µl of experimental culture medium into 96-well microtiter plates (TPP, Techno Plastic Products AG, Trasadingen, Switzerland). The plates were incubated for 4 h to allow cell adhesion. Subsequently, 20 µl of experimental culture medium containing increasing concentrations of berbamine or cisplatin, respectively, were added to each well. Cisplatin was added to the berbamine-treated samples of RT112 LTT and J82 LTT at defined concentrations. After 48 h of incubation, 20 µl of CellTiter-Blue® reagent (Promega, Mannheim, Germany) were added. This reagent contains resazurin, a blue dye that penetrates viable cells and is reduced by intracellular redox enzymes to the fluorescent pink compound resorufin. Resorufin can diffuse out of the cells into the surrounding medium. After 2.5 h of incubation at room temperature in the dark, fluorescence intensity was measured using a microplate reader (FLUOstar Optima, BMG Labtech, Ortenberg, Germany) at an excitation/emission wavelength of 560/590 nm. Data analysis was performed (OPTIMA v2.0, BMG Labtech). Relative cell viability (%) was calculated by normalizing the fluorescence intensities of each sample to the mean of the corresponding triplicate wells without berbamine and cisplatin. Background fluorescence from control wells containing only medium and corresponding concentrations of berbamine or cisplatin was subtracted beforehand. From these data, IC_{50} values and fitted curves were determined by analysis with a statistical software (GraphPad Prism v10.2.3) using the provided equation *log(inhibitor) vs. response - Variable slope (four parameters)*.

Results

Treatment with berbamine or cisplatin resulted in a concentration-dependent decline in cell viability, which followed a sigmoidal curve across all tested cell lines (Supplementary Fig. S 4). The highest IC_{50} cisplatin values were detected in the resistant variants RT112 LTT (43.7 µM) and J82 LTT (30.7 µM), confirming successful induction of cisplatin resistance. As specified by the cell line donor, both cell lines were routinely cultured and treated under selective pressure with 51.5 µM and 8.3 µM cisplatin, respectively. In contrast, their corresponding parental lines exhibited lower IC_{50} cisplatin values: 28.6 µM for RT112 and 6.8 µM for J82. The CSCs demonstrated the greatest sensitivity to cisplatin, with IC_{50} values of 18.9 µM for RT112 CSC and 3.7 µM for J82 CSC. Interestingly, RT112 CSC, J82, and J82 CSC showed a more gradual response to increasing cisplatin concentrations, as reflected by shallower concentration-response slopes. RT112 cell line variants exhibited naturally higher resistance to cisplatin compared to J82 variants.

Berbamine treatment resulted in comparable IC_{50} values across all cell lines, ranging from 7.7 µM in RT112 LTT to 13.6 µM in RT112 CSC. Intermediate values were observed for RT112 (10.7 µM), J82

CSC (11.0 µM), J82 (11.3 µM), and J82 LTT (13.5 µM). Notably, RT112 LTT, which was continuously cultured under high-dose cisplatin treatment, exhibited the lowest IC_{50} berbamine. All cell lines showed a steep decline in cell viability in response to berbamine, with curves approaching zero across the tested concentration range. With the exception of J82 and J82 CSC, the IC_{50} berbamine values were consistently lower than those for cisplatin, suggesting comparable or greater cytotoxic potency under the tested conditions.

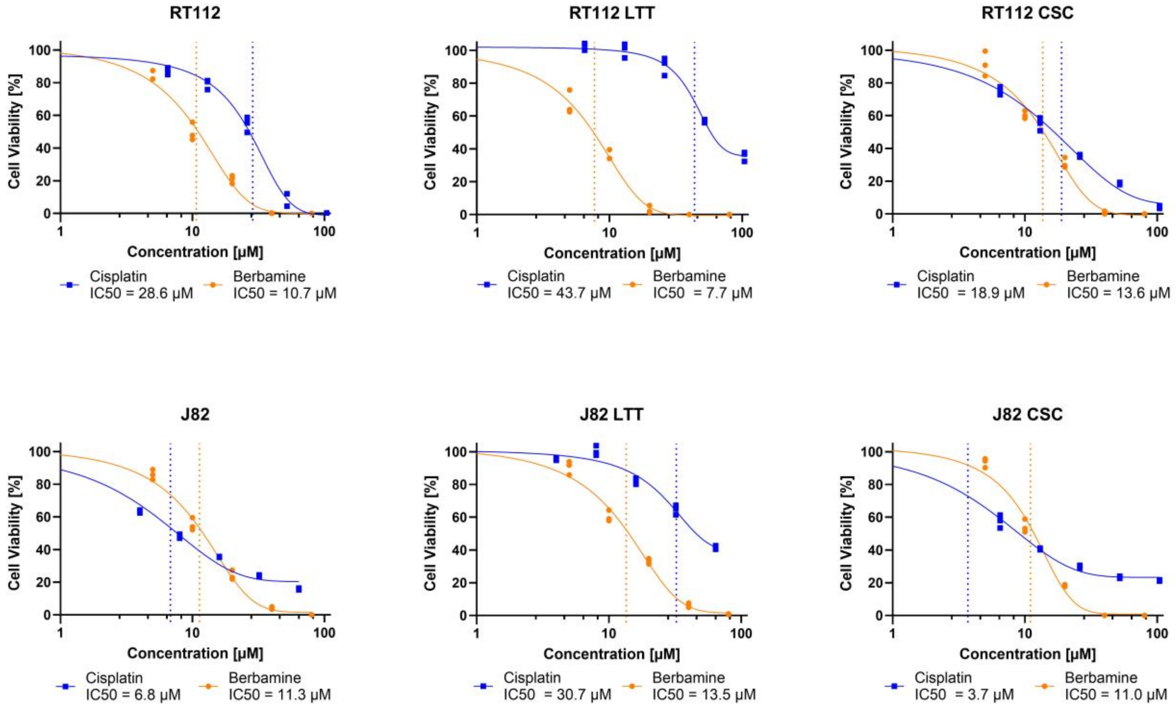

Supplementary Fig. S 4: Cell viability of RT112 and J82 cell line variants following 48-hour treatment with increasing concentrations of cisplatin (blue) or berbamine (orange). Cell viability of RT112, RT112 LTT, RT112 CSC (top), and J82, J82 LTT, J82 CSC (bottom) was assessed using the CellTiter-Blue® assay. Data are presented as single values of percentage to the untreated control (n = 3). Nonlinear regression analysis was performed to determine the fitted curves and IC_{50} values, which are depicted as horizontal dotted lines for each graph.

3.2. For combination treatment with 5-ALA-PDT and berbamine, and analysing ROS production

Materials and methods

The previously described protocol for IC_{50} determination of berbamine was adapted to the experimental setup of combination treatment with 5-ALA-PDT and berbamine, as well as for analysing reactive oxygen species (ROS) production. This was done to ensure accurate correlation between berbamine concentration and cell viability. Cells were seeded at a density of 1.5×10^4 cells per well in experimental culture medium. Based on the doubling time of 24 h (Supplementary Fig. S 3), RT112 were seeded at half the standard density, with 7.5×10^3 cells per well, for RT112 LTT with a doubling time of 60 h, a higher seeding density of 22.5×10^3 cells per well was used. On day two and after 24 h of incubation, 50 µl of the same medium containing various concentrations of berbamine were added to each well. Following another 24-hour incubation, the medium was aspirated and replaced with 200 µl of fresh DMEM/F12 containing the same concentrations of berbamine. To assess potential interaction effects between berbamine and 5-ALA (Fagron, Rotterdam, Netherlands) on cell viability, half of the wells were additionally treated with 5-ALA of 100 µg/ml. 5-ALA was previously dissolved in PBS, pH-neutralized to 7.4 using NaOH (Carl Roth, Karlsruhe, Germany), and sterile-filtered through a 0.22 µm membrane (Merck, Darmstadt, Germany). From the time of 5-ALA exposure, all further steps were carried out in the dark. At day four and after a total 48 h berbamine exposure, the medium was again removed and replaced by 100 µl of DMEM/F12 without phenol red (ThermoFisher) or berbamine but 10% FBS and 1 mM sodium pyruvate. On the fifth day, 20 µl of CellTiter-Blue® reagent was added,

and fluorescence intensity was measured. Data analysis and calculations were performed analogously to the initial IC_{50} berbamine determination. To determine the final IC_{50} berbamine, the mean of the IC_{50} values obtained from conditions with and without 5-ALA treatment was calculated.

Results

Under the experimental conditions used for both combination treatment and ROS detection via 5-ALA-PDT, IC_{50} berbamine values were found to differ slightly from previous measurements. As shown in Supplementary Fig. S 5, co-treatment with 5-ALA of 100 µg/ml resulted in lower or equal IC_{50} berbamine values in all cell lines except J82, when compared to berbamine monotherapy. For subsequent experiments, the mean IC_{50} berbamine values derived from treatments with and without 5-ALA were calculated for each cell line. The lowest IC_{50} berbamine values were observed in the cisplatin-resistant variants RT112 LTT (7.5 µM) and J82 LTT (5.9 µM), followed by their parental lines RT112 (9.5 µM) and J82 (10.7 µM). The cancer stem cells RT112 CSC (10.3 µM) and J82 CSC (13.6 µM) showed the highest IC_{50} berbamine in their variant group. All cell lines exhibited sigmoidal dose-response curves that approached zero cell viability at the highest tested berbamine concentrations. Notably, in RT112 LTT, RT112 CSC, and J82 LTT, the decline in cell viability was less steep under 5-ALA co-treatment, resulting in lower IC_{50} berbamine values compared to treatment without 5-ALA.

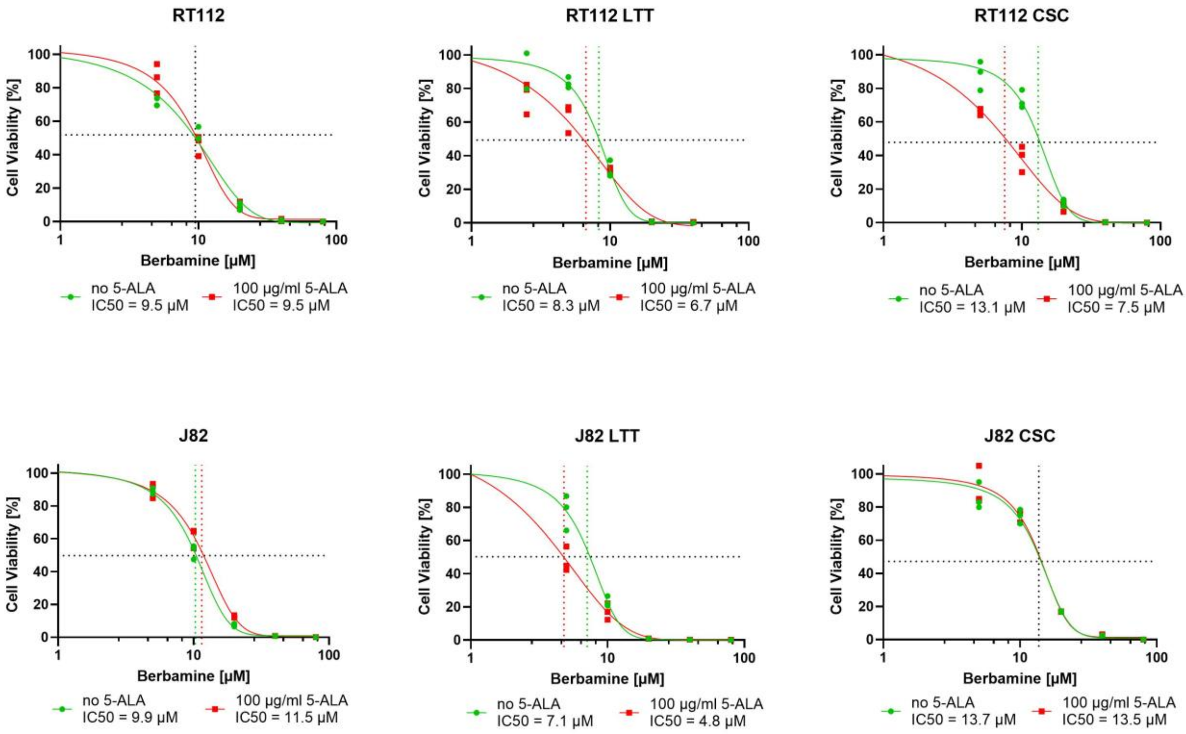

Supplementary Fig. S 5: Cell viability of RT112 and J82 cell line variants with increasing berbamine concentrations in the absence (green) or presence (red) of 5-ALA of 100 µg/ml. Cell viability of RT112, RT112 LTT, RT112 CSC (top), and J82, J82 LTT, J82 CSC (bottom) was assessed using the CellTiter-Blue® assay. Data are presented as single values of percentage to untreated control (n = 3). Nonlinear regression analysis was performed to determine the fitted curves and IC_{50} berbamine values, which are depicted as horizontal dotted lines for each graph.

4. Characterization of bladder cancer cell properties in relation to berbamine

4.1. Cell migration

Materials and methods

Cell migration behaviour in response to berbamine was evaluated in a wound healing assay using specialized 24-well microtiter plates equipped with silicone inserts (Ibidi, Gräfelfing, Germany). These inserts create a defined cell-free gap of 500 µm ± 100 µm within the confluent cell monolayer. Cells were seeded at a density of 4 to 5 × 10^5 cells/ml to ensure confluency after 24 h. A volume of 70 µl of

the cell suspension in experimental culture medium was pipetted into each of the two compartments of the silicone inserts. The plates were then incubated for at least 24 h. Once a confluent monolayer had formed, the silicone inserts were carefully removed using sterile tweezers, and the wells were washed with 500 µl of experimental culture medium to remove any non-adherent cells. Then, 1 ml of medium containing ¼ IC_{50}, ½ IC_{50}, and IC_{50} berbamine concentrations, as well as berbamine-free controls, was added to each well in triplicate. Plates were incubated again at 37 °C and 5% CO_2, and images were digitally captured (CamLabLite™) every 3 h at 40× magnification until the gap in the control group was closed entirely by migrating cells, using an inverted light microscope (Leica DM IL) equipped with a USB digital camera (MikrOkular Full HD). Gap closure was further measured by an image analysis software (ImageJ v2.14.0, National Institutes of Health, Bethesda, MD, USA; plugin *in vitro scratch wound assay*), and the quantitative data were then graphically displayed using a statistical software (GraphPad Prism v10.2.3).

Results

Berbamine influenced the migratory capacity of the bladder cancer cell lines to varying degrees (Supplementary Fig. S 6). The fast proliferating RT112 cells showed no response to berbamine treatment, even at the highest concentration tested. In all RT112 samples, the gap was fully closed within 42 h. In contrast, RT112 CSC cells demonstrated reduced migration under high-dose treatment. Lower concentrations led to complete gap closure within 54 h, whereas the IC_{50}-treated sample required approximately 72 h to reach a comparable level of closure. RT112 LTT and J82 CSC untreated and low-dose samples (¼ IC_{50} berbamine in J82 CSC) achieved almost complete closure after 72 h, whereas gaps in IC_{50}-treated samples remained open throughout the observation period. J82 and J82 LTT initially showed no difference in migration across treatment groups for the first 10 h. After this point, migration rates began to diverge: control samples and low-dose treatments (¼ IC_{50} berbamine in J82) reached full closure after 42-45 h. In J82 LTT, however, treatment with ½ IC_{50} and IC_{50} berbamine not only inhibited migration but also led to gap widening over time, accompanied by visible cell shrinkage and detachment under phase-contrast microscopy, indicative of cytotoxic effects.

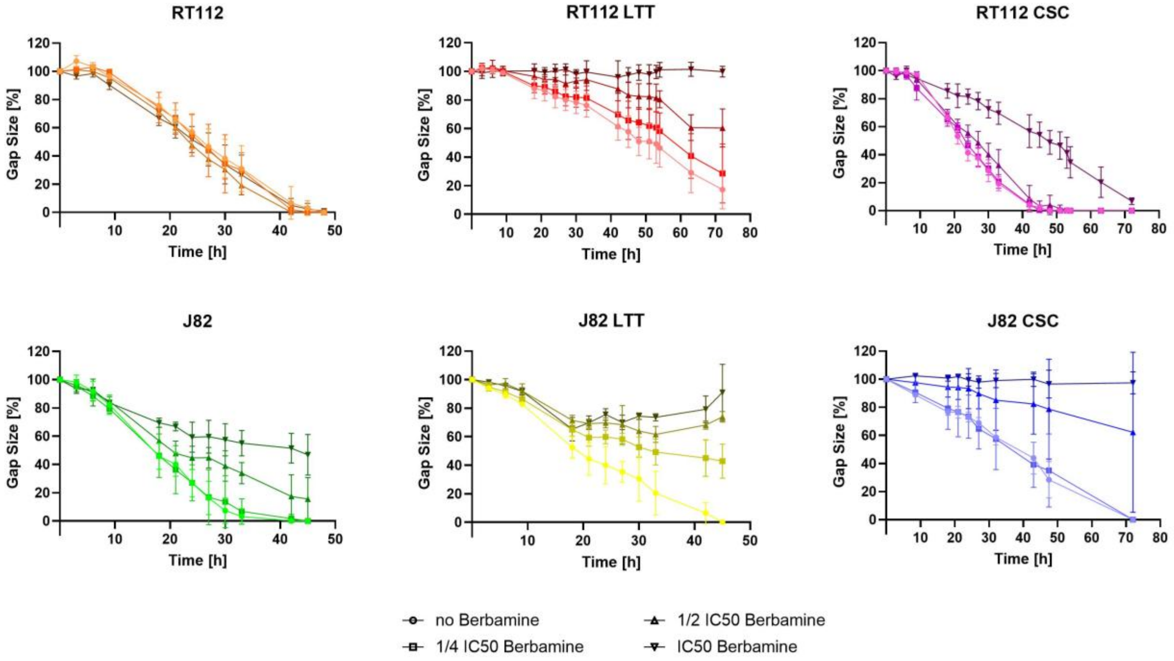

Supplementary Fig. S 6: Berbamine impairs cell migration in RT112 and J82 cell line variants. Wound healing assays were performed for RT112, RT112 LTT, RT112 CSC (top), and J82, J82 LTT, J82 CSC (bottom) using berbamine (no, ¼ IC_{50}, ½ IC_{50} and IC_{50}). Gap closure [%] was monitored every 3 h until complete closure in the untreated control and quantified using an image analysis software (ImageJ v2.14.0). Data represent mean ± SD (n = 3) and are connected with a simple line to guide the eye.

4.2. Cell invasion

Materials and methods

Cell invasion in response to berbamine was assessed and quantified using a Boyden chamber system consisting of Matrigel-coated inserts placed in 24-well companion plates (Corning). The inserts were first coated with ice-cold, liquid growth factor-reduced Matrigel Basement Membrane Matrix (Corning), prepared by diluting 30 µg of Matrigel in 100 µl of DMEM GlutaMAX™ per insert. The plates were then incubated for at least 4 h at 37 °C to allow the Matrigel to solidify. To determine the optimal seeding density, preliminary experiments were performed using increasing cell numbers per insert in 250 µl of serum-free DMEM GlutaMAX™. The following densities were defined as optimal: RT112 and J82 (6×10^4), J82 CSC (8×10^4), RT112 CSC and J82 LTT (1×10^5), RT112 LTT (2×10^5). After incubation, residual liquid was removed from the Matrigel layer and the determined optimal cell number was then seeded into each insert in triplicates. Treatment conditions included berbamine-free controls and berbamine at ¼ IC_{50}, ½ IC_{50}, and IC_{50} concentrations, all in serum-free medium. Cisplatin was added to the cisplatin-resistant cell lines at defined concentrations. The lower chamber of each well was filled with DMEM GlutaMAX™ supplemented with 10% FBS and 1 mM sodium pyruvate, with the FBS serving as a chemoattractant. Following 48-hour incubation, cells that had invaded through the Matrigel-coated insert and adhered to the underside of the membrane were fixed using 4% paraformaldehyde (Roti-Histofix; Carl Roth) and stained with 1% crystal violet (Merck). For quantification, three representative regions per insert were captured at 100× magnification (CamLabLite™) to reflect overall cell density, using an inverted light microscope (Leica DM IL) equipped with a USB digital camera (MikrOkular Full HD). Image analysis was performed (ImageJ v2.14.0), and data visualisation was carried out by statistical software (GraphPad Prism v10.2.3). To enable graphical comparability between cell lines, cell counts are shown as the number of invaded cells per image, normalised to 10^3 seeded cells per insert.

Results

The data presented in Supplementary Fig. S 7 illustrate cell line-specific differences in the ability to invade through a Matrigel-coated membrane toward FBS as a chemoattractant, as well as the inhibitory effect of berbamine at previously determined IC_{50} concentrations. RT112, J82, and J82 LTT displayed comparable invasive behaviour under control conditions, which decreased markedly following berbamine treatment. Invasion decreased by 49% in RT112, by 65% in J82 CSC, and by 75% in J82 at IC_{50}. RT112 CSC exhibited approximately half the invasive potential of their parental cell line under control conditions and experienced a modest 28% reduction upon berbamine treatment. The cisplatin-resistant lines RT112 LTT and J82 LTT showed the lowest basal invasion in the absence of berbamine. Of all the tested cell lines, J82 LTT exhibited the strongest response to berbamine, with invasion decreasing by over 82% at IC_{50} berbamine. RT112 LTT, however, showed a moderate reduction of 44%.

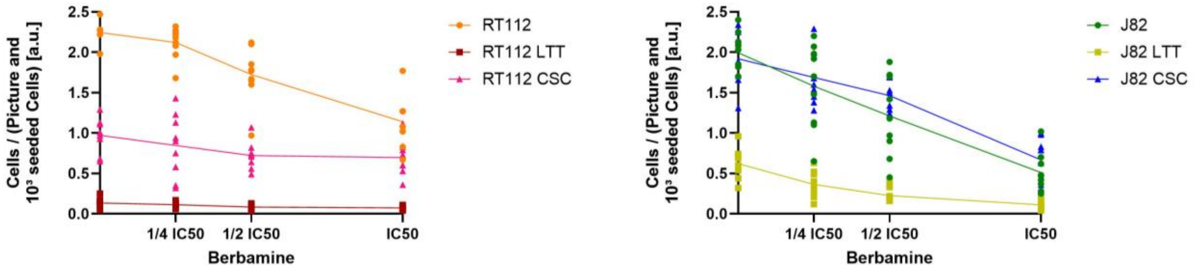

Supplementary Fig. S 7: Berbamine impairs cell invasion in RT112 and J82 cell line variants. Matrigel invasion assays were performed for RT112, RT112 LTT, RT112 CSC (left), and J82, J82 LTT, J82 CSC (right) treated for 48 h with berbamine (no, ¼ IC_{50}, ½ IC_{50}, and IC_{50}). Data represented as single values ($n = 9$) together with connection of the mean values with a simple line to guide the eye.

4.3. Apoptosis rate

Materials and methods

To investigate apoptosis induced by berbamine, 2 × 10^5 cells were seeded in experimental culture medium in 25 cm^2 culture flasks. Following 24 h of incubation, ½ IC_{50} or IC_{50} berbamine was added. A control flask was maintained without berbamine treatment. The culture medium for cisplatin-resistant cell lines was supplemented with cisplatin at defined concentrations. On day 5 after berbamine addition, the cells were harvested using 1% trypsin-EDTA, centrifuged at 472 × g for 4 minutes, and resuspended in 2 ml PBS. An aliquot of 500 µl was transferred into each of three Fluorescence-activated cell sorting (FACS) tubes (alpha laboratories, Eastleigh, Hampshire, UK). The triplicates were washed with 500 µl PBS containing 1% FBS, centrifuged at 472 × g for 4 minutes, and resuspended in 200 µl of FVS575V working solution. The stock solution consisted of 200 µg FVS575V (Becton Dickinson, San Jose, CA, USA) dissolved in 340 µl dimethyl sulfoxide (DMSO) and was diluted 1:1000 in PBS to prepare the final working solution. The cells were incubated for 15 minutes at room temperature in the dark. Following incubation, the cells were washed twice with PBS containing 1% FBS, centrifuged again, and resuspended in 100 µl of Annexin V binding buffer (diluted 1:10 with H_2O; Becton Dickinson). Next, 5 µl of Annexin V-allophycocyanin (Annexin-APC; Becton Dickinson) was added, and the cells were incubated for another 15 minutes in the dark at room temperature. After adding 100 µl of Annexin V binding buffer, apoptosis was measured within one hour using a flow cytometer (FACSCelesta™; Becton Dickinson).
Dual staining enabled discrimination between early apoptosis, late apoptosis, and necrosis. Early apoptotic cells were identified by Annexin V-APC binding to externalized phosphatidylserine (PS) in a Ca^{2+}-dependent manner. APC was excited using the red laser (627-640 nm) and detected with a 660 nm bandpass filter. To distinguish early apoptotic from late apoptotic and necrotic cells, FVS575V was applied. This cell viability dye covalently binds to cellular amines and penetrates cells with compromised membranes. FVS575V was excited by the violet laser (396 nm) and emitted fluorescence at 572 nm. Flow cytometry data acquisition (FACSDiva™; Becton Dickinson) and analysis (FlowJo software v9.9.5, LLC, Ashland, OR, USA) were conducted (Supplementary Fig. S 8), and further statistical analysis and graphical representation (GraphPad Prism v10.2.3) were performed.

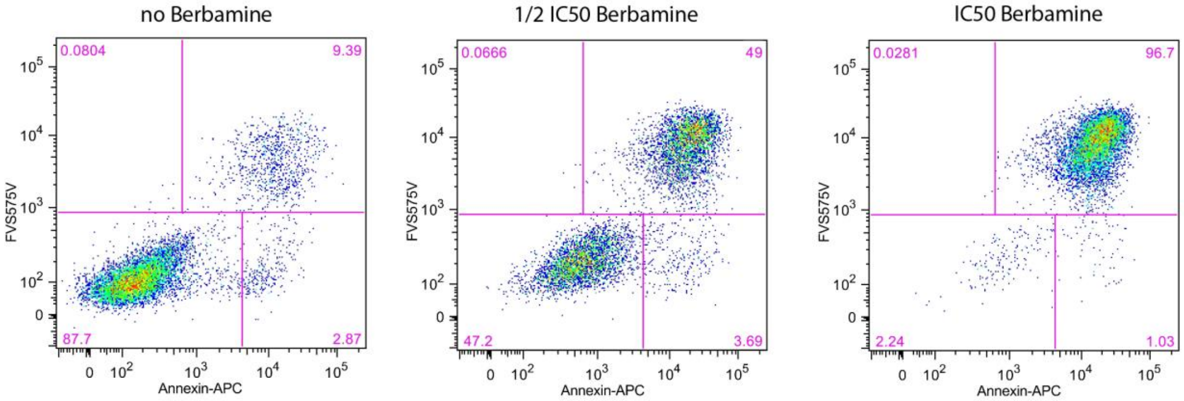

Supplementary Fig. S 8: Representative flow cytometry plots of apoptosis induction by berbamine in J82 LTT cells. Dot plots show Annexin V-APC and FVS575V staining of J82 LTT cells treated with berbamine (no, ½ IC_{50}, and IC_{50}) for 5 days. Percentages: viable cells in lower left quadrant; early apoptotic cells in lower right quadrant; late apoptotic/necrotic cells in upper right quadrant.

Results

Berbamine treatment led to increased cell death in all tested bladder cancer cell lines (Supplementary Fig. S 9). In the RT112 cell line variants, a higher proportion of dying cells in early apoptosis was measured, in contrast to the J82 cell line variants, which showed a greater shift toward late apoptosis and necrosis. Notably, in RT112 cells, the proportion of early apoptotic cells remained high across all berbamine concentrations. For RT112 and RT112 CSC, no statistically significant increase in the total percentage of dead cells (sum of early and late apoptosis and necrosis) was noted at ½ IC_{50} berbamine compared to the untreated control. At IC_{50}, the proportion of dead cells increased to 52% in RT112 and

43% in RT112 CSC. In RT112 LTT, cell death reached 67%, accompanied by a decrease in early apoptotic and an increase in late apoptotic and necrotic cells. J82 cell variants exhibited greater sensitivity to berbamine, with highly significantly elevated ($p < 0.0001$) apoptosis rates at IC_{50}. The total proportion of dead cells was 57% in J82, 73% in J82 CSC, and 97% in J82 LTT, indicating a markedly increase in vulnerability under these experimental conditions. Notably, the extent of cell death observed for J82 LTT in the apoptosis assay exceeded that seen in the original cell viability-based IC_{50} berbamine determinations.

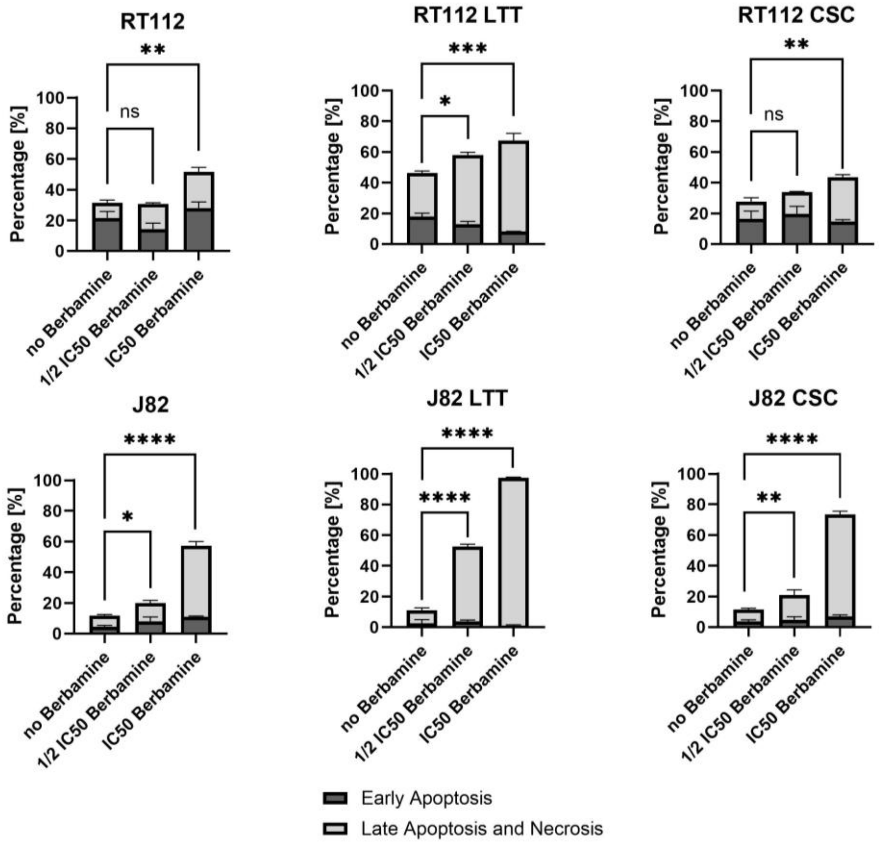

Supplementary Fig. S 9: Berbamine induces apoptosis in RT112 and J82 cell line variants. Apoptosis was analysed in RT112, RT112 LTT, RT112 CSC (top), and J82, J82 LTT, J82 CSC (bottom) after treatment with berbamine (no, ½ IC_{50}, and IC_{50}) for 5 days. Early apoptosis and late apoptosis/necrosis were detected via Annexin V-APC and FVS575V staining followed by flow cytometry. Data represent mean ± SD (n =3). Asterisks indicate significance of the total apoptotic and necrotic cell fractions relative to the control without berbamine. (*$p < 0.05$, **$p < 0.01$, ***$p < 0.001$, ****$p < 0.0001$).

5. Determination of ROS generation

To evaluate berbamine- or 5-ALA-PDT-induced ROS generation, the *Cellular Reactive Oxygen Species Detection Assay Kit* by Abcam (Cambridge, UK) was used. The cell-permeant 2′,7′-dichlorofluorescin diacetate (DCFDA; Abcam) gets deacetylated intracellularly by esterases and subsequently oxidized by ROS to the fluorescent compound 2′,7′-dichlorofluorescein (DCF).

5.1. Determination of ROS generation following treatment with berbamine

Materials and methods

For investigation of ROS generation following treatment with berbamine, 2.5 × 10^4 cells/well were seeded into black, clear-bottom 96-well microtiter plates (Corning) in 100 µl experimental culture medium. Due to differences in cell doubling times among the tested cell lines, RT112 cells were seeded at 12.5 × 10^3 cells per well, while RT112 LTT cells were seeded at 37.5 × 10^3 cells per well. Each cell line was plated in triplicates for seven treatment conditions. Plates were incubated overnight at 37 °C and 5% CO_2. On the following day, cells were washed once with 100 µl of assay buffer (Abcam). Subsequently, cells were incubated with 100 µl of freshly prepared 20 µM DCFDA in assay buffer for 45 minutes at 37 °C in the dark. To account for background fluorescence, a set of wells was treated with

DCFDA-free medium. After incubation, the DCFDA solution was removed, and wells were washed twice with 100 µl of assay buffer. Cells were then treated with berbamine (no, ¼ IC_{50}, ½ IC_{50}, IC_{50}, and 2× IC_{50}) diluted in phenol red-free DMEM/F12 with 10% FBS and 1 mM sodium pyruvate. For positive control, cells were treated with 150 µM tert-butyl hydroperoxide (TBHP; Abcam), as this molecule provokes oxidative stress and ROS generation intracellularly. All treatments were performed for 4 h at 37 °C in the dark. DCF fluorescence intensity was measured using a microplate reader (FLUOstar OPTIMA) at excitation/emission wavelengths of 485/520 nm. Data were analysed with integrated (OPTIMA v2.0, BMG Labtech) and statistical (GraphPad Prism v10.2.3) software. Fluorescence intensities from DCFDA-free wells were subtracted to correct for background signal. Relative ROS levels were expressed as fold change compared to berbamine-untreated controls incubated with 20 µM DCFDA.

Results

Berbamine monotreatment (that means without the influence of a 5-ALA-PDT) resulted in minimal or no increase in ROS generation, depending on the cell line (Supplementary Fig. S 10). In RT112 LTT, J82, and J82 CSC, no statistically significant changes in ROS production were detected across all tested berbamine concentrations, including up to 2× IC_{50}. RT112 CSC even showed a modest but significant reduction ($p < 0.05$) in ROS production following berbamine exposure. RT112 demonstrated a significant increase ($p < 0.01$) in ROS generation at 2× IC_{50} berbamine. However, since all other functional assays in this study were performed using berbamine concentrations up to IC_{50}, this observation is not directly relevant for the interpretation of those results. Among all cell lines, J82 LTT was the only one to exhibit a significant increase ($p < 0.01$) in ROS generation already at IC_{50} berbamine, suggesting cell line-specific responses to berbamine with respect to ROS induction.

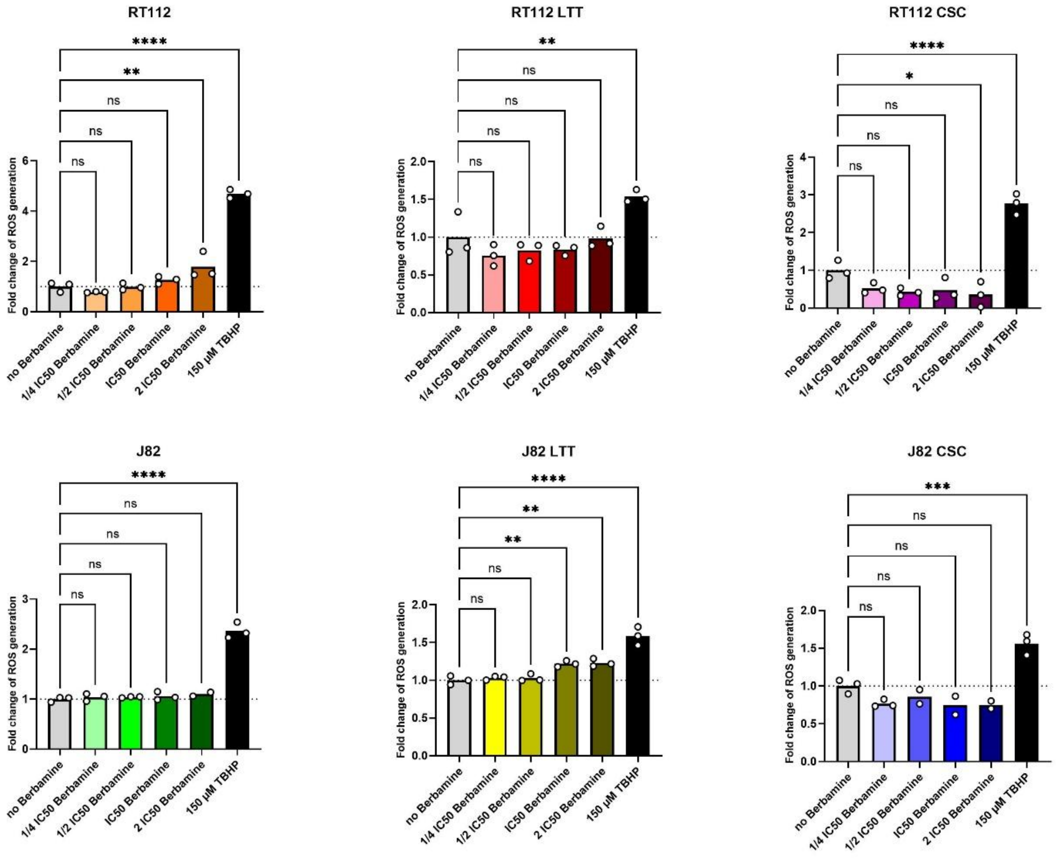

Supplementary Fig. S 10: Effect of berbamine on ROS generation in RT112 and J82 cell line variants. RT112, RT112 LTT, RT112 CSC (top), and J82, J82 LTT, J82 CSC (bottom) were treated with berbamine (no, ¼ IC_{50}, ½ IC_{50}, IC_{50}, and 2× IC_{50}) for 4 h. ROS levels were measured using the DCFDA assay and expressed as fold change relative to the berbamine-untreated controls. TBHP (150 µM) served as positive control. Data are presented as single values (n = 3) and mean. (*$p < 0.05$, **$p < 0.01$, ***$p < 0.001$, ***$p < 0.0001$).

5.2. Determination of ROS generation following 5-ALA-PDT

Materials and Methods

The ROS detection protocol was adapted to the specific requirements of the combination treatment involving 5-ALA-PDT and berbamine, in order to accurately investigate the levels of ROS generated under these treatment conditions. First, cells were seeded into black, clear-bottom 96-well microtiter plates at a density of 1.5×10^4 cells per well in 150 µl of experimental culture medium. To account for differences in cell doubling rates, RT112 cells were plated at 7.5×10^3 cells per well, and RT112 LTT cells at 22.5×10^3 cells per well. Each of the seven treatment conditions was performed for six different irradiation doses in six replicates. On the second day and after 24 h incubation, 50 µl of experimental culture medium was added to each well. For the cisplatin-resistant cell lines, cisplatin was added in predefined concentrations. On the third day, the medium was aspirated, and freshly prepared 5-ALA was added at final concentrations of 0, 25, 50, or 100 µg/ml in serum-free DMEM/F12. The 5-ALA stock solution was previously dissolved in PBS, adjusted to pH 7.4 using NaOH, and sterile-filtered through a 0.22 µm membrane. From the moment of 5-ALA exposure, all subsequent procedures were performed under light-protected conditions. On the fourth day, cells were washed and stained with 20 µM DCFDA in phenol red-free DMEM/F12 with 10% FBS and 1 mM sodium pyruvate, except DCFDA-negative control wells, and incubated for 45 minutes at 37 °C. DCFDA was removed, and wells were washed twice with assay buffer. 100 µl of phenol red-free DMEM/F12 with 10% FBS and 1 mM sodium pyruvate were added to the cells, which were then exposed to photodynamic treatment using red light (635 ± 3nm) at an intensity of 100 mW/cm² for irradiation times of 0, 20, 40, 80, 160, or 320 seconds, corresponding to light doses of 0, 2, 4, 8, 16, and 32 J/cm², respectively. Irradiation was applied from below in a custom-built chamber equipped with a temperature-controlled platform, ensuring homogeneous light distribution across the cell plane and maintaining a constant temperature of 37 °C during light exposure [43]. For positive control wells, 200 µM tert-butyl hydroperoxide (TBHP) in phenol red-free DMEM/F12 were added for 4 h. Negative control samples with DCFDA and no DCFDA were not irradiated, either. Immediately following 5-ALA-PDT or TBHP incubation, DCF fluorescence intensity was measured using a microplate reader (FLUOstar OPTIMA) at excitation/emission wavelengths of 485/520 nm. Raw fluorescence intensity data were analysed using integrated software (OPTIMA v2.0) and further processed in statistical software (GraphPad Prism v10.2.3). Background fluorescence from cell-free and DCFDA-negative wells was subtracted from all values. ROS induction was expressed as fold change compared to the non-irradiated control treated with DCFDA.

Results

5-ALA-PDT led to increased ROS generation in all tested bladder cancer cell lines (Supplementary Fig. S 11). At 5-ALA levels of 100 µg/ml, all cell lines showed highly significant increases ($p < 0.0001$) in ROS levels compared to the irradiated control without 5-ALA. Highly significant ($p < 0.0001$) ROS induction was also noted at 5-ALA concentrations of 50 µg/ml in all cell lines except for J82 LTT, and a modest significant increase ($p < 0.05$) for RT112 CSC. At 25 µg/ml, no significant differences from the irradiated control were detected, and for RT112, ROS production was even modestly but significantly reduced ($p < 0.05$). RT112 exhibited the strongest ROS response under 5-ALA-PDT conditions: At the highest irradiation doses (16 and 32 J/cm²), ROS levels approached those of the positive control treated with 200 µM TBHP. Notably, the TBHP control of RT112 yielded the lowest ROS induction compared to the 5-ALA-free irradiated control among all cell lines, which may partially explain the comparability in this context. In contrast, the ROS generation of the irradiated samples of all other cell lines remained below 50% of the ROS levels induced by TBHP, even at maximum conditions. RT112 CSC showed particularly low sensitivity, reaching only 30% of the ROS signal compared to the TBHP control. This cell line, however, was the only one to exhibit a continuous increase in ROS generation, whereas all other cell lines showed saturation at 16 J/cm² or even at 8 J/cm² in the case of J82 LTT and J82 CSC.

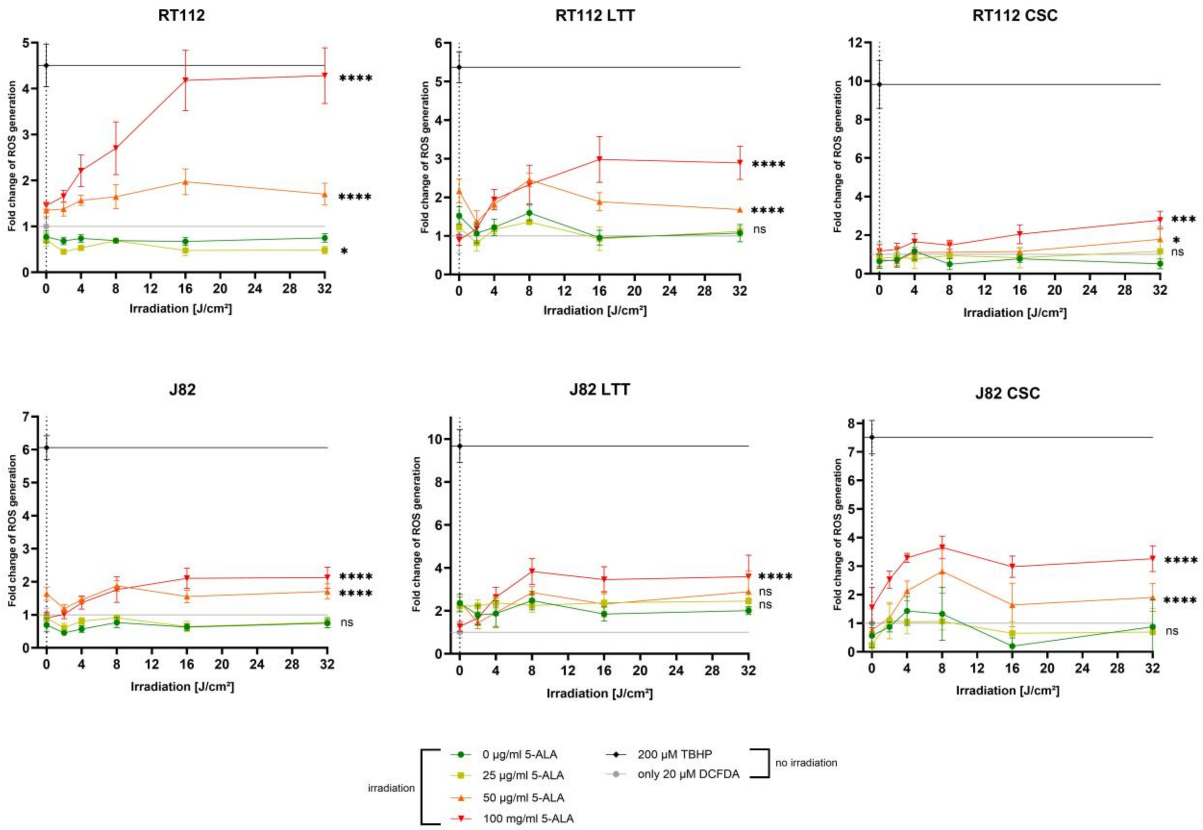

Supplementary Fig. S 11: ROS generation following 5-ALA-PDT in RT112 and J82 cell line variants. RT112, RT112 LTT, RT112 CSC (top) and J82, J82 LTT, J82 CSC (bottom) were treated with 5-ALA (0-100 µg/ml) and exposed to increasing doses of red light (635 ± 3nm, 0-32 J/cm²). ROS production was quantified using the DCFDA assay and expressed as fold change relative to untreated controls. TBHP (200 µM, no light) served as positive control. Data are shown as mean ± SD (n = 6). Asterisks indicate significance compared to the irradiated control without 5-ALA (*p < 0.05, **p < 0.01, ***p < 0.001, ***p < 0.0001).

6. Statistical analysis

All statistical analyses were performed using the statistical software (GraphPad Prism v10.2.3). Where appropriate, the numerical data were expressed as means ± standard deviation (SD). Differences between groups were assessed by two-way ANOVA followed by two-tailed Dunnett's post hoc analysis. A significance threshold of p = 0.05 was applied. Due to partially small sample size, normality testing was limited; however, the tests were applied based on visual distribution checks and variance homogeneity, as they are considered robust under such conditions.
